# Integrating machine learning for the identification of ubiquitination-associated genes in moyamoya disease

**DOI:** 10.3389/fneur.2025.1653433

**Published:** 2025-09-16

**Authors:** Hongchuan Niu, Xilong Wang, Zhenyu Zhou, Yutong Liu, Shihao He, Yuanli Zhao

**Affiliations:** ^1^Department of Neurosurgery, Peking University International Hospital, Beijing, China; ^2^Department of Neurosurgery, Beijing Tiantan Hospital, Capital Medical University, Beijing, China; ^3^Department of Neurosurgery, Peking Union Medical College Hospital, Peking Union Medical College and Chinese Academy of Medical Sciences, Beijing, China

**Keywords:** moyamoya disease, epigenetics, ubiquitination, machine learning, immune infiltration

## Abstract

**Introduction:**

Moyamoya disease (MMD) is an infrequent cerebrovascular disorder typified by bilateral internal carotid artery obstruction, yet its pathogenic mechanism remains elusive. This study examines the role of epigenetic ubiquitination-related genes in MMD.

**Methods:**

We utilized two datasets (GSE157628 and GSE141024) from the GEO database and sourced ubiquitination-related genes from the GeneCards database. Differentially expressed genes were identified, followed by Gene Ontology (GO), Kyoto Encyclopedia of Genes and Genomes (KEGG), and Gene Set Enrichment Analysis (GSEA) to elucidate key gene functions. Machine learning techniques, including LASSO logistic regression and support vector machine, helped identify crucial genes. Immune characteristics were analyzed using single-sample gene set enrichment analysis, while transcription factors and miRNA-gene regulatory networks were constructed with the Citrome and Mircode databases.

**Results:**

We identified three key ubiquitination-related genes—ANAPC11, UCHL1, and USP41—that may be involved in the pathogenesis of MMD. Further, we found that the serum UCHL1 expression level in MMD was significantly reduced, and knocking down UCHL1 could enhance the migration ability of human brain vascular smooth muscle cells (HBVSMCs), as verified by *In vitro* experiments. Immune infiltration analysis demonstrated significant correlations between these genes and various immune factors. Furthermore, we constructed a miRNA-gene network involving 30 miRNAs and identified secondary genes EXO1 and ISG15.

**Discussion:**

Potential therapeutic drugs, including benzohydroxamic acid and PKC-beta inhibitors, were predicted to target these key genes, suggesting new avenues for MMD treatment.

## Introduction

Moyamoya disease (MMD) is a rare cerebrovascular condition where the terminal part of the internal carotid arteries (ICAs) experiences stenosis or obstruction, leading to the development of an abnormal vascular network at the base of brain, consisting of vulnerable perforating vessels (1). Epidemiological investigations have revealed that MMD is more prevalent among East Asians compared to Westerners and that it occurs more frequently in women (2). MMD seizures show two age peaks: childhood and young adulthood (2). However, the pathogenesis of MMD is still unclear (2, 3). Current research has indicated that both genetic and microenvironmental factors contribute to its development (3–5).

In recent years, researchers have discovered that mutations in a single gene cannot adequately account for the onset of MMD. A growing body of evidence underscores the regulatory function of epigenetic markers in pivotal cellular and molecular processes associated with the pathogenesis of MMD (6, 7). Epigenetics typically encompasses DNA methylation, histone modifications, and non-coding RNAs. To date, genome-wide approaches to analyzing the epigenome have gradually expanded the observed epigenetic features in MMD.

The ring finger protein 213 (*RNF213*) gene located in the 17q25-ter region has been identified as the primary susceptibility gene for MMD in East Asian populations (8, 9). Specific variations leading to single-nucleotide polymorphisms (SNPs) (preponderantly p. R4810K and p. R4859K) in RNF213 have been associated with the familial and sporadic cases in Japanese, Korean, and Chinese (8, 9). Subsequent studies have reported the discovery of several other new variants of *RNF213* identified in non-p. R4810K East Asian and Caucasian patients (8, 10, 11). RNF213 is a 591-kDa cytosolic protein with two functional domains: a Walker motif and a RING finger domain exhibiting dual AAA + adenosine triphosphatase (ATPase) domains and ubiquitin ligase activities (8). A study further revealed the complex folding of the mouse RNF213 including an amino-terminal (N-terminal) stalk, a core structure reminiscent of dynein featuring six ATPase units, and a complex E3 ubiquitin ligase module with multiple domains by scanning its structure with the cryogenic electron microscopy (cryo-EM) (12). This study also suggested that pathological MMD mutations are concentrated in the E3 domain and might interfere with substrate ubiquitination (12). In addition, another study indicated that mutations in the RING finger domain of RNF213 reduced its ubiquitin ligase activity and enhanced the activation and apoptosis of nuclear factor kappa B (NF-kappa B) in an AAA + domain-dependent manner, and that this dysregulation might contribute to MMD pathogenesis (13). Consequently, it is easy to hypothesize ubiquitination modification may play a crucial role in the development of MMD. However, there is a lack of research on the mechanism of ubiquitination modification in MMD, and the genetic factors associated with ubiquitination in MMD remain understudied.

In addition to directly pathogenic genetic factors, the complex microenvironmental factors also be involved in the pathogenesis of MMD (3, 4). The composition of the microenvironment is very complex. Several researches have explored the specific landscape of immune cell infiltration in MMD (14, 15). For example, Li et al. reported that the abundances of eosinophils, natural killer T (NKT) cells, and T helper (Th) 2 cells were significantly different between patients with MMD and controls (14). Another study recently conducted by Cao et al. investigated that eosinophils were highly proportioned in the specific immune infiltration landscape of MMD, rather than T or B cells (15). However, the precise proportions of various immune cells present in MMD require future research.

Thus, our research aims to identify gene factors associated with ubiquitination modification in MMD, analyze the relationship between key ubiquitination-related genes and immune cell infiltration, and explore the potential molecular mechanisms underlying the occurrence of MMD. Additionally, we analyzed and depicted the miRNA-gene regulatory network, and predicted potential targeted therapeutic drugs.

## Materials and methods

### Downloading and processing of data

Gene Expression Omnibus (GEO) (https://www.ncbi.nlm.nih.gov/geo/) (accessed on 12 August 2023) database was the source of our data. We downloaded the series matrix files of GSE157628 dataset from this database. These files contain 20 transcription profiles collected from 11 MMD patients and 9 age- and sex-matched controls. Additionally, we obtained the series matrix files of GSE141024 dataset from GEO database which consist of eight transcription profiles collected from four MMD patients and four matched controls. The annotated file for the both datasets is GPL16699.

We then used the GeneCards database (https://www.genecards.org/) to extract ubiquitination-related genes. Relevance score is a metric used in the GeneCards database to measure the relevance of a gene to a specific topic or field. This score is calculated based on a variety of data sources and information resources. Specifically, a higher relevance score indicates a stronger correlation between a gene and the topic or field. We set a screening condition that only genes with a relevance score > 10 were selected to ensure that the genes we selected had high relevance to ubiquitination.

### Batch effect correction and analysis of differentially expressed genes (DEGs)

After merging and normalizing the datasets, we utilized the Surrogate Variable Analysis (SVA) package in R software to perform batch effect correction on the data. Subsequently, we plotted the sample distributions before and after correction using principal component analysis (PCA) to visualize how the batch effect changed before and after correction. The “limma” package in R software was used to examine the distinctions in molecular mechanisms between MMD and control samples, with the objective of pinpointing DEGs. The selection criteria were based on an absolute log fold change (FC) exceeding 0.585 and a *p*-value below 0.05. Additionally, we plotted volcano and clustering heat maps to visualize the results.

### Functional enrichment analysis of gene ontology (GO) and Kyoto encyclopedia of genes and genomes (KEGG)

A comprehensive functional enrichment analysis was performed on the differentially expressed ubiquitination-related genes using the Metascape database (https://www.metascape.org). Relevant functional categories were evaluated through the application of GO and KEGG pathways. Statistical significance was only achieved if minimum overlap ≥3 and *p* ≤ 0.01. And we plotted histograms and network diagrams to visualize the results.

### Feature selection process for least absolute shrinkage and selection operator (LASSO)logistic regression and support vector machine (SVM) algorithms

Our study integrated the LASSO logistic regression and SVM algorithms to select the key genes of MMD (16). LASSO logistic regression, a linear regression approach applied to select features in a model by incorporating a penalty term that restricts the coefficients within the model, forcing the coefficients of certain features to zero, thereby enabling automatic feature selection, and is capable of efficiently handling high-dimensional datasets, reducing overfitting, and identifying the most relevant features. In our study, LASSO logistic regression algorithms were implemented using the “glmnet” package within R software. We modeled gene expression data using the “glmnet” function, specifying a Gaussian distribution (“family” set to “gaussian”), and determined the optimal lambda value through a rigorous 10-fold cross-validation process. The key parameter of LASSO regression, lambda, which serves as the coefficient of the penalty term, determines the degree to which the LASSO regression model constrains feature selection, with larger values causing more feature coefficients to converge to zero, thus selecting fewer features, whereas smaller values result in more features entering the model, thereby increasing its complexity. To determine the optimal lambda value, a 10-fold cross-validation was conducted. The classification effectiveness of the model was assessed using the “type.measure = ‘class’“parameter within the “cv.glmnet” function. Then, the optimal lambda value corresponding to “lambda.min” was selected to avoid overfitting and improve the generalization ability and stability of the model. We plotted the performance curves for each lambda value, and by examining the curve’s nadir, we determined the optimal lambda value to identify the featured genes.

Support vector machine-recursive feature elimination (SVM-RFE) is an algorithm for feature selection based on SVMs that progressively removes unimportant features by recursion and retains the features that contribute most to the classification task. The basic idea of the method is to construct a classification model using SVM and calculate the weight of each feature, followed by gradually removing features with smaller weights until the remaining set of features is optimal. In this study, we implemented the SVM-RFE method with the “e1071” software package in R software and combined it with cross-validation to evaluate the performance of the model to identify biomarkers with high diagnostic value. In SVM-RFE, the number of feature selection iterations (i.e., starting from the initial set of features and gradually eliminating unimportant features until the best subset of features remains) has a significant impact on the final set of genes selected. To guarantee the robustness of the feature selection procedure and to gauge how the removal of features in each cycle affects model efficacy, we employed 5-fold cross-validation. Cross-validation helped us select the optimal subset of features such that these features exhibited the best predictive performance on all training datasets. We used the “e1071” package based on SVM-RFE method to select the features of differential genes to get the ranking of each gene, and to utilize the top 10 ranked genes to construct SVM models. Then we evaluated the minimum point of error rate of the models and get the combination of genes when the error rate reached the minimum point.

LASSO regression can perform feature selection while handling multicollinearity, whereas SVM-RFE focuses on evaluating the importance of features using support vector machine algorithms. The combination of these two algorithms can manage feature selection for both high-dimensional data and nonlinear relationships, thereby enhancing the overall performance of feature selection. Furthermore, it can also improve the model’s generalization ability. Therefore, the combined application of these two machine learning methods aims to enhance the stability of the model and the accuracy of feature selection in this study. Finally, by combining the genes derived from the SVM-RFE and LASSO algorithms, we aimed to pinpoint the key genes and assess their diagnostic value for MMD.

### *In vitro* experimental verification

The validation set consists of self-test data from peripheral venous blood samples from 3 MMD patients and 3 healthy controls (HCs). The collection of biospecimens and data was approved by the Institutional Ethics Committee of Peking Union Medical College Hospital, Beijing, China (I-24PJ2435), and all specimens were obtained with written informed consent.

All blood samples were collected in the morning and rapidly centrifuged at 2500 rpm for 10 min at 4°C to obtain the supernatant as serum. The serum was then transferred to a new EP tube and stored at −80°C. After thawing, the level of UCHL1 were measured using Human UCHL1 ELISA Kit (HUES03248) (AssayGenie, Dublin, Ireland) according to the manufacturer’s instructions.

Commercial Human brain vascular smooth muscle cells (HBVSMCs) (ScienCell Research Laboratories, Hubei, China) were subcultured in SMCM medium containing 1% P/S bispecific antibody, 1% smooth muscle cell growth supplement (SMCGS), and 2% FBS. When the cells fused to 90%, the old medium was discarded and the cells were washed twice with 2 mL PBS. After discarding PBS, 2 mL of 0.25% trypsin 0.02% EDTA mixed digestion solution was added and observed under a microscope for about 30 s. When the cells became round, 2 mL of complete medium was quickly added to terminate digestion, gently blown, and collected. 800 rpm, Centrifuge at 4°C for 5 min, discard the supernatant, resuspend the cells in complete culture medium, culture in separate bottles, and change the medium the next day. On the day before transfection, approximately 2.5 × 105 cells were seeded in a 6-well plate and incubated for 24 h, resulting in a cell density of approximately 60–70%. Use Lipofectamine 3,000 transfection reagent (L3000150) (Thermo Fisher Scientific, Pittsburgh, PA, USA) to transfect HBVSMC with siRNA. Incubate at 37 ° C in a 5% CO2 incubator for 4–6 h, then replace with fresh medium and incubate for 3 days.

After transfection, prepare each group as a cell suspension and seed them into 6-well plates, with 5 × 10^5^ cells per well. When the cells have fused to 90%, perform the corresponding treatments according to the experimental groups. After treatment, digest the cells, centrifuge, precipitate, dry, and dissolve to obtain the total RNA solution. Take 2 μL of the total RNA as the template, prepare the reverse transcription reaction system according to the kit instructions, place the reaction system in the PCR instrument, select the preset program, and react at 25°C for 5 min, 46°C for 20 min, and 95°C for 1 min to obtain cDNA, which is stored at −70°C. Prepare the reaction system and qPCR reaction conditions according to the protocol instructions, and perform amplification according to the pre-set program. The obtained data, after processing, represent the mRNA expression levels.

Based on the experimental groups, each group was prepared as a cell suspension and seeded into 6-well plates, with 1 × 10^6^ cells per well. Upon reaching 90% confluence, the supernatant was discarded, and the cell samples were washed twice with pre-chilled PBS. For every 100 μL of cell sample, 1 mL of RIPA containing PMSF was added, and the cells were lysed thoroughly. The lysate was then centrifuged at 4°C at 12,000 × *g* for 5 min. The supernatant was immediately transferred to a pre-chilled Eppendorf tube, representing the extracted cellular protein, and stored at −80°C for later use. Protein quantification was conducted using the BCA method. Finally, 5 × loading buffer was added, and the sample was heated in a boiling water bath for 10 min. The sample preparation was complete and could be stored at −20°C. Depending on the molecular weight of the target protein, prepare a 10% separating gel and a 5% stacking gel. Cast SDS-PAGE gels, adding 5 mL of prepared separating gel to each gel plate. After adding the gel, apply isopropanol and press the gel. Once the gel lines have formed (gel set), place the gel rack horizontally and remove the isopropanol with filter paper. After mixing the prepared stacking gel, inject 2 mL of the prepared stacking gel at the top of the separating gel. Immediately insert the comb vertically, ensuring it remains horizontal during insertion. After the stacking gel has solidified, remove the comb and place it in the electrophoresis chamber containing the electrophoresis buffer. Add an appropriate amount of pre-chilled 1 × electrophoresis buffer, then begin loading the samples. Perform constant-voltage electrophoresis at 80 V for approximately 30 min. Once the samples have entered the separating gel, adjust the voltage to 120 V and continue electrophoresis. Stop electrophoresis once the target band reaches the desired position. Cut the PVDF membrane to match the size of the gel, activate it in methanol for 1 min, then soak it in the transfer buffer. Place the filter paper in the transfer buffer and soak for 15 min. Assemble the transfer “sandwich” following the principle of PVDF membrane ≥ gel ≥ filter paper, ensuring all bubbles are removed before starting constant-pressure transfer. After transfer is complete, stain the membrane with Lithmus Red S solution, then wash twice with TBST and observe the proteins on the membrane. Wet the membrane with TBS from bottom to top, then transfer it to a dish containing a blocking solution (5% non-fat milk powder in TBST) and shake at room temperature on a shaking incubator for 1 h to block the immunoglobulin binding sites on the PVDF membrane. Wash the membrane with TBST to remove residual liquid, then place the membrane in a self-sealing bag using a sealer, seal three sides, add the primary antibody diluted to the appropriate concentration with TBST, expel air bubbles as much as possible, seal the bag opening, and incubate at 4°C overnight. Open the self-sealing bag, wash the membrane three times with TBST, each for 10 min. Then place the membrane in a sealing bag, add an appropriate amount of secondary antibody at the appropriate concentration, seal the bag, and incubate at room temperature for 1 h. Open the self-sealing bag, wash the membrane three times with TBST, each for 10 min. Mix equal volumes of chemiluminescent reagent A and B, and place the membrane protein side down in contact with the mixture. After 5 min, detect using the Tanon 5,200 chemiluminescence imaging workstation. If multiple exposures are required for the same PVDF membrane, wash with strip solution (protein blot membrane regeneration solution) at room temperature for 45 min, then wash with TBST three times, and restart from the blocking step, with subsequent steps as before; Protein expression levels were analyzed using Image Pro Plus 6.0 software to assess optical density values. Relative protein expression was calculated as the ratio of target protein gray value to internal control protein gray value, and phosphorylated protein gray value to total protein gray value.

Cell scratch assay was used to test the migration ability of HBVSMC. Use a UV disinfected marker pen to evenly draw horizontal lines behind the 6-well plate, approximately every 0.5–1 cm, crossing the holes with at least 5 lines per hole. Cells in logarithmic growth phase are digested into single-cell suspension using trypsin, and the concentration of the cell suspension is adjusted by adding appropriate culture medium. 2 mL of cells with a density of 6 × 105 cells/mL are seeded into a 6-well plate marked with lines to ensure full cell growth the next day. The final total amount of culture medium per well is 2 mL. The cells are cultured in a 37°C, 5% CO2 cell culture incubator according to the grouping requirements. After 24 h, when observing the cell adhesion and uniform distribution under a microscope, use a disinfected 200 *μ* L gun head to scratch the marked horizontal line on the back in a super clean bench, with the gun head vertical and not tilted. Wash the cells three times with PBS, remove the cut cells, add serum-free medium, and culture in a 37°C, 5% CO2 incubator. Take a photo at a magnification of 100 times, ensuring that the scratch is centered and vertical, paying attention to consistent background, sampling according to time points, and taking photos for recording.

### Analysis of immune cell infiltration

Single-sample gene set enrichment analysis (ssGSEA) represents a commonly employed technique for evaluating the types of immune cells present in the microenvironment. We used the ssGSEA algorithm in the “GSVA” package of R software to quantify 29 human immune cell phenotypes in MMD, and infer the relative proportion of immune-infiltrating cells. The Spearman’s rank correlation coefficient was used to correlate gene expression with the immune cells. And we plotted cell content histograms, violin plots, and cell correlation heat maps to visualize the results.

### Gene set enrichment analyses (GSEA)

GSEA, respectively, divides the samples into high- and low-expression groups based on the median expression value of key genes. The genes were categorized based on the level of differential expression between the two groups. Subsequently, an analysis was conducted to determine if the background gene datasets were concentrated at either the top or bottom of this categorized list. The datasets labeled c2.cp.kegg (version 7.0) were sourced from the MsigDB database (https://www.gsea-msigdb.org/gsea/msigdb/index.jsp) and served as the background gene datasets for this study. To delve deeper into the molecular mechanisms underlying key genes, we employed GSEA (https://www.gsea-msigdb.org/gsea/index.jsp) to analyze and compare the disparities in KEGG signaling pathways between samples from the high-expression and low-expression groups. Subsequently, we arranged the gene datasets that were significantly enriched (adjusted *p* < 0.05) based on their concordance scores.

### Prediction of a MicroRNA (miRNA)-genes regulatory network

Cistrome database (http://cistrome.org/db/) which contains a large number of samples related to chromatin immunoprecipitation sequencing (ChIP-seq) and deoxyribonucleasednase-sequencing (DNase-seq), was used to explore the regulatory interactions between transcription factors and key genes. In this study, the genome file was configured to use the hg38 reference, with the transcription start site designated as 10 kb upstream. The obtained results were then graphically represented using Cytoscape software, facilitating a clearer understanding and analysis. MiRNAs are small non-coding RNAs that regulate gene expression by promoting messenger RNAs (mRNAs) degradation or inhibiting translation. We utilized the miRcode database (http://www.mircode.org/) to identify miRNAs related to the key genes and employed the Cytoscape software to illustrate the networks of these miRNAs.

### Prediction of potential drugs using connectivity map (CMap)

CMap (https://www.broadinstitute.org/connectivity-map-cmap) is a comprehensive gene-expression profiling database established by the Broad Institute. It encompasses a vast array of gene microarray data, specifically detailing the responses of 1,309 small molecule drugs before and after treatment in five distinct human cell lines, all examined under a wide range of conditions. This resource serves as a valuable tool for researchers seeking to understand the molecular mechanisms underlying drug action and response. This study identified potential therapeutic agents for MMD by targeting the key genes. L1000 as a background dataset provided us with contextual information on perturbation response patterns and helped us to more accurately identify the effects of drugs on gene expression, thus revealing the functional link between drugs and MMD.

### Statistical analysis

In bioinformatics analysis, all statistical computations were performed utilizing the R software package (version 4.2.2). The statistical tests conducted were two-tailed, with statistical significance set at *p* < 0.05. During the self-test validation experiments, the data were examined and visualized using Graphpad Prism 9 (Version 9.4.0), and subsequently organized and illustrated using Adobe Illustrator 2022 (Version 26.3.0). The data are presented as mean values ± standard deviation (SD), and statistical variations among groups were evaluated using the t-test and one-way ANOVA. A *p*-value of less than 0.05 is regarded as indicative of statistical significance.

## Results

### DEGs identification and functional enrichment analysis

We selected and downloaded the GSE157628 and GSE141024 datasets related to MMD from the GEO database, including expression profiling data from 28 patients (The control group comprised 13 participants, while the MMD group had 15). GSE157628 consisted of 11 patients with MMD and nine controls, and GSE141024 consisted of four patients with MMD and four controls.

**Figure 1:**
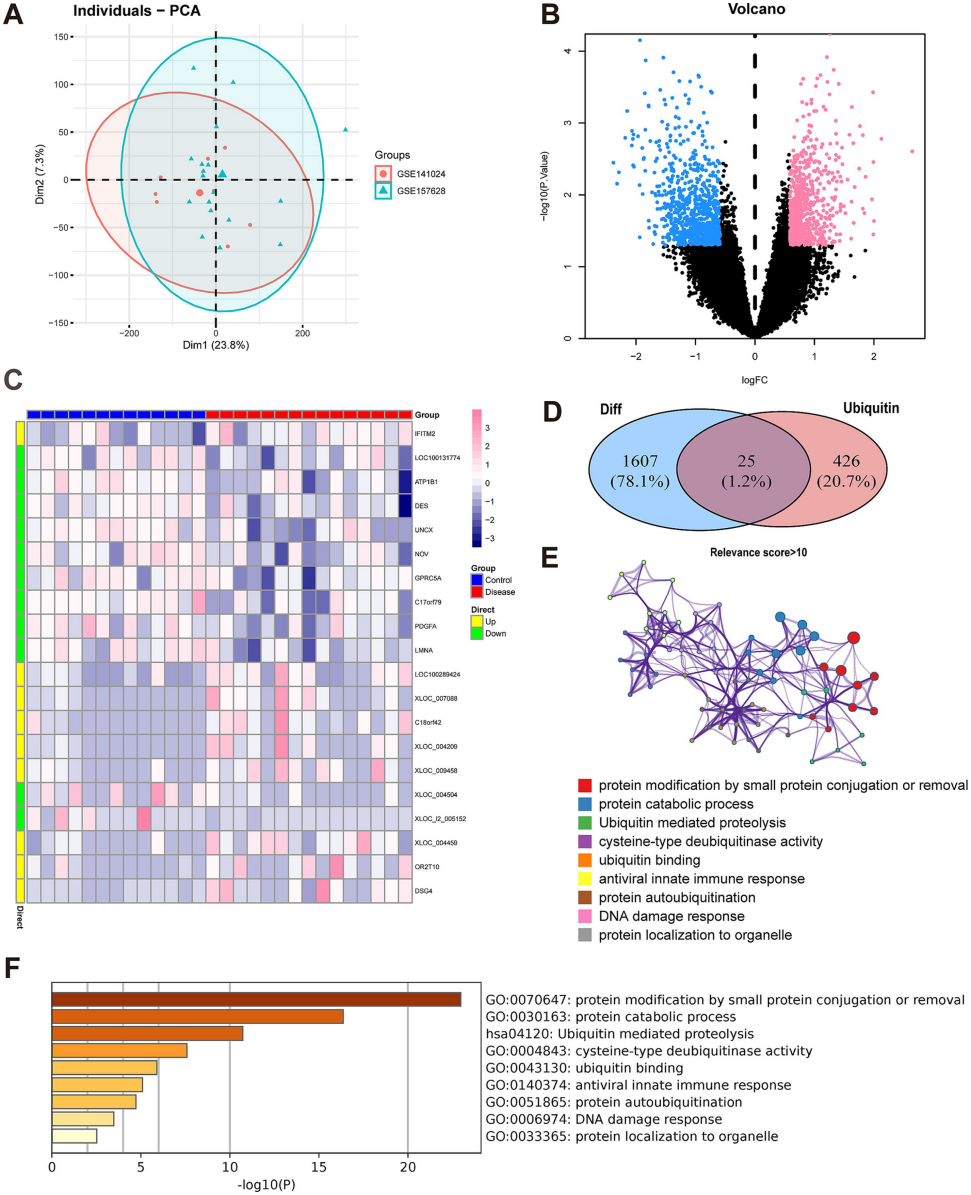
Differentially expressed gene (DEG) analysis and functional annotation. **(A)** Principal component analysis (PCA). The scatterplot depicts the results of PCA between the GSE157628 and GSE141024 datasets. **(B)** DEG analysis. A volcano plot is utilized to present the differentially expressed genes (DEGs). In the MMD group, down-regulated genes are indicated by blue dots, whereas up-regulated genes are represented by red dots. Black dots signify genes that are not differentially expressed. **(C)** DEG analysis. A heat map is employed to display the expression patterns of DEGs. Each column corresponds to a sample, and each row corresponds to a DEG. **(D)** The identification of differentially expressed ubiquitination-related genes was carried out. A Venn diagram illustrates 25 ubiquitination-related genes with differential expression in MMD. **(E)** Functional annotation. A histogram shows the top nine signaling pathways that the 25 genes were mainly enriched to. The horizontal axis (X) represents the enrichment degree, while the vertical axis (Y) signifies the regulatory pathway. **(F)** Function annotation. A network indicates the relationship between these signaling pathways. The dot color indicates the pathways. The size of the dots indicates the degree of pathway enrichment.

After integrating and standardizing the data, we employed the SVA package within the R software framework to address and mitigate batch effects. We plotted the sample distributions before (Supplementary Figure S1) and after correction using PCA (Figure 1A) to illustrated that the batch effects between different microarrays were significantly reduced after correction.

Using the “limma” package, we identified 1,632 differentially expressed genes (Figures 1B,C). Based on the criteria of |logFoldChange(FC)|exceeding 0.585 and a *p*-value below 0.05, the expression levels of 794 genes were found to be upregulated, while those of 838 genes were downregulated.

Subsequently, ubiquitination-related genes with relevance scores > 10 were extracted from the GeneCards database. These ubiquitination-related genes and the DEGs described above were taken to intersect and we obtained 25 differentially expressed ubiquitination-related genes (Figure 1D).

In addition, we conducted a functional enrichment analysis of the aforementioned genes utilizing the Metascape database. Both GO and KEGG were employed to evaluate the pertinent functional categories. Statistical significance was only achieved if minimum overlap ≥ 3 and *p* ≤ 0.01. These differentially expressed ubiquitination-related genes were predominantly enriched in signaling pathways, such as ubiquitin mediated proteolysis, protein catabolic process and ubiquitin binding (Figures 1E,F).

### Selection of the key genes of MMD

**Figure 2:**
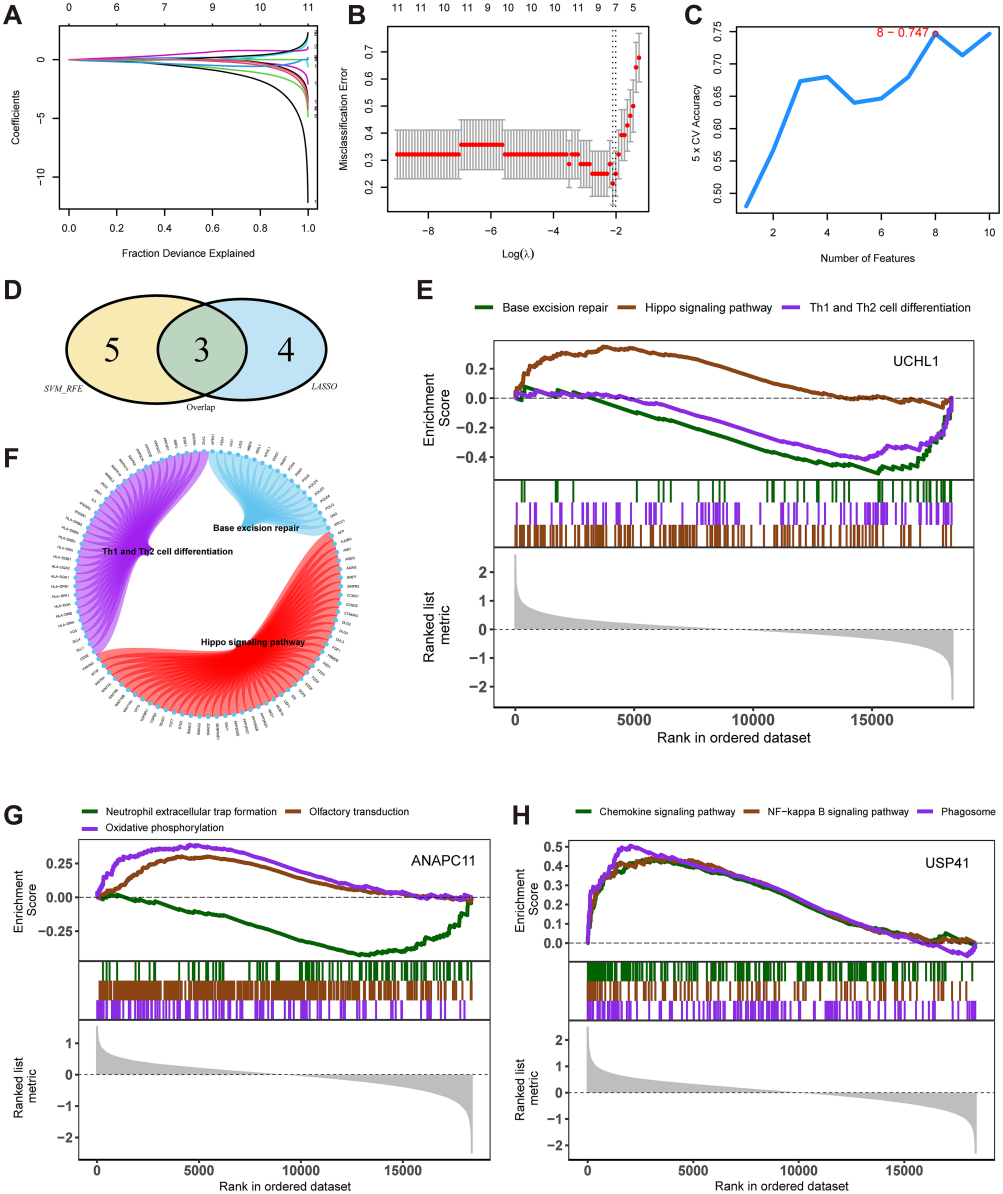
Selection of the key genes of Moyamoya disease (MMD) and enrichment analysis of key genes. **(A)** Least absolute shrinkage and selection operator (LASSO) regression was employed. Feature selection was carried out using the LASSO regression model. The coefficient variations of different genes were observed with different lambda values (fraction deviance explained). **(B)** LASSO regression. To determine the optimal parameter (lambda) for the LASSO model, a graph was plotted of the partial likelihood deviance (binomial deviance) curve against log (lambda) for validation purposes. **(C)** Analysis with support vector machine (SVM) algorithm. In the support vector machine - recursive feature elimination method, the accuracy of the model varied as the number of features changed. **(D)** A Venn plot shows three key genes of MMD. **(E)** Signaling pathways enrichment analysis. UCHL1 was enriched in three major pathways. **(F)** A Circos plot shows the pathways enriched by UCHL1. **(G)** Signaling pathways enrichment analysis. ANAPC11 was enriched in three major pathways. **(H)** Signaling pathways enrichment analysis. USP41 was enriched in three major pathways.

To conduct feature selection among the 25 pre-selected differentially expressed ubiquitination-related genes, we combined the LASSO logistic regression and SVM algorithms. The LASSO regression algorithm identified seven feature genes in MMD (Figures 2A,B), and the SVM-RFE algorithm showed that eight feature genes had the highest accuracy when screening the MMD datasets (Figure 2C). By taking the intersection, we obtained three key genes from these two sets of feature genes: anaphase promoting complex subunit 11 (*ANAPC11*), ubiquitin carboxyl-terminal hydrolase L1 (*UCHL1*), and ubiquitin specific peptidase 41 (*USP41*) (Figure 2D).

### *In vitro* experimental verification

**Figure 3:**
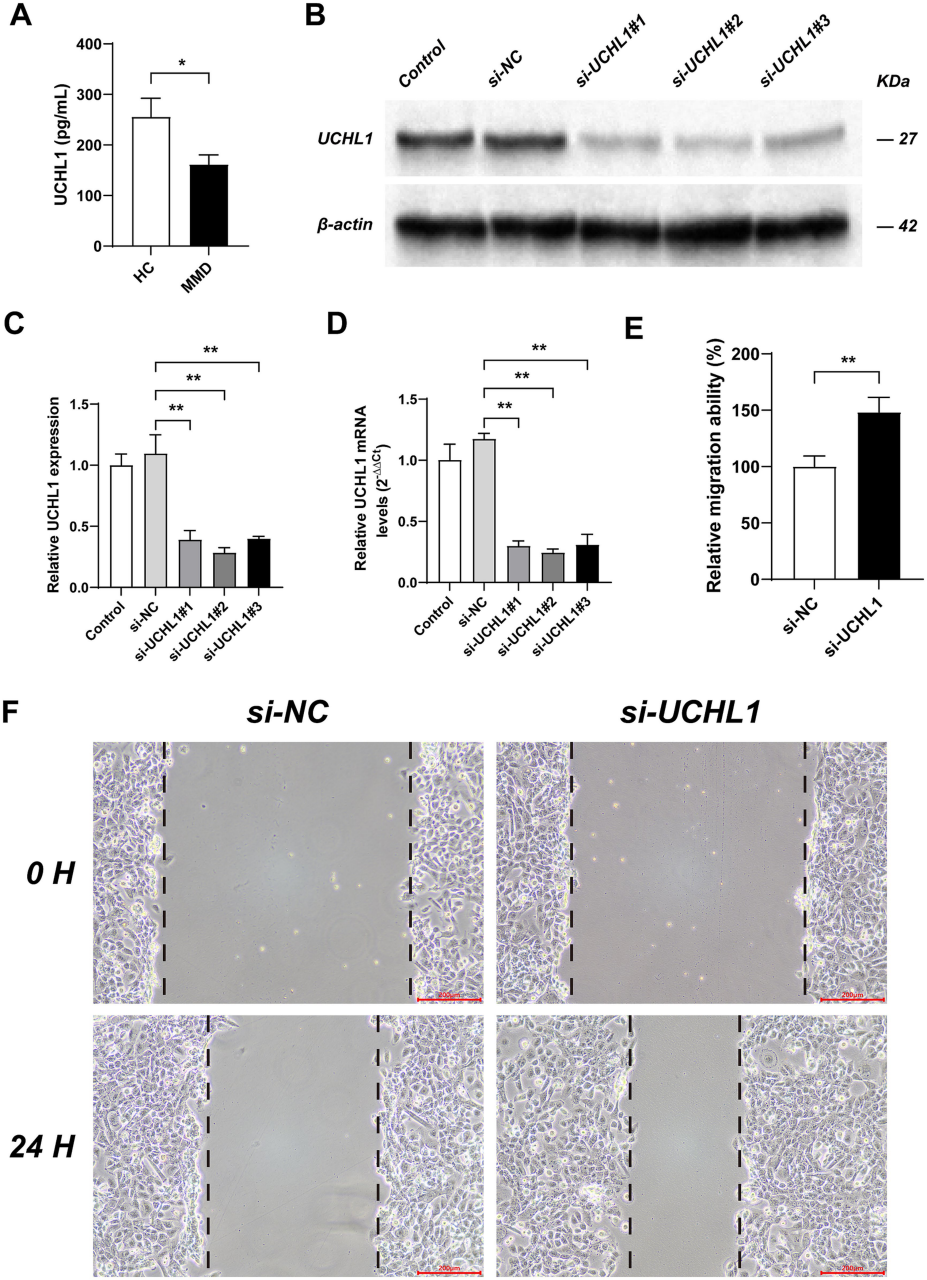
The expression level of UCHL1 in MMD and the effects of UCHL1 gene knockout on vascular smooth muscle migration ability. **(A)** The contents of UCHL1 in the serum were measured by ELISA assay. **(B)** The Western Blot experiment results showed the transfection efficiency of si-RNAs in HBVSMC. **(C)** Results of qPCR analysis of the transfection efficiency of si-RNAs. **(D)** Results of qPCR analysis of the transfection efficiency of si-RNAs based on relative quantitative method. **(E)** The bar chart shows the effects of UCHL1 gene knockout on the migration ability of HBVSMC. **(F)** Cell migration was detected by scratch assay. Bar = 200 micrometers. Results were mean ± SD for all individual experiments. *p < 0.05; **p < 0.01.

We utilized the Human UCHL1 ELISA Kit to measure the expression level of UCHL1 and discovered that, compared with the HC group, the serum UCHL1 content in the MMD group was significantly reduced (Figure 3A).

Subsequently, we employed si-NC (SS: UUCUCCGAACGUGUCACGUTT, AS: ACGUGACACGUUCGGAGAATT); si-UCHL1#1 (SS: 5’-GGACAAGAAGUUAGUCCUAAA -3′, AS: 5′- UAGGACUAACUUCUUGUCCCU -3′); si-UCHL1#2 (SS: 5′- GGUUUCUGUCUGUAAGUUAAG -3′, AS: 5′- UAACUUACAGACAGAAACCAA −3′); and si-UCHL1#3 (SS: 5′- GUUUCUGUCUGUAAGUUAAGA -3′, AS: 5′- UUAACUUACAGACAGAAACCA −3′) to transfect HBVSMC and validated the transfection efficiency using Western Blot experiments and PCR (Figures 3B–D). The results indicated that the transfection efficiency of si-UCHL1#2 was the highest, and it was chosen for the subsequent cell scratch assay.

The cell scratch assay was conducted to assess the migration ability of HBVSMC (Figures 3E,F). The results demonstrated that, compared with the si-NC group, the migration ability of HBVSMC cells in the si-UCHL1 group was significantly enhanced.

### Analysis of immune cell infiltration

**Figure 4:**
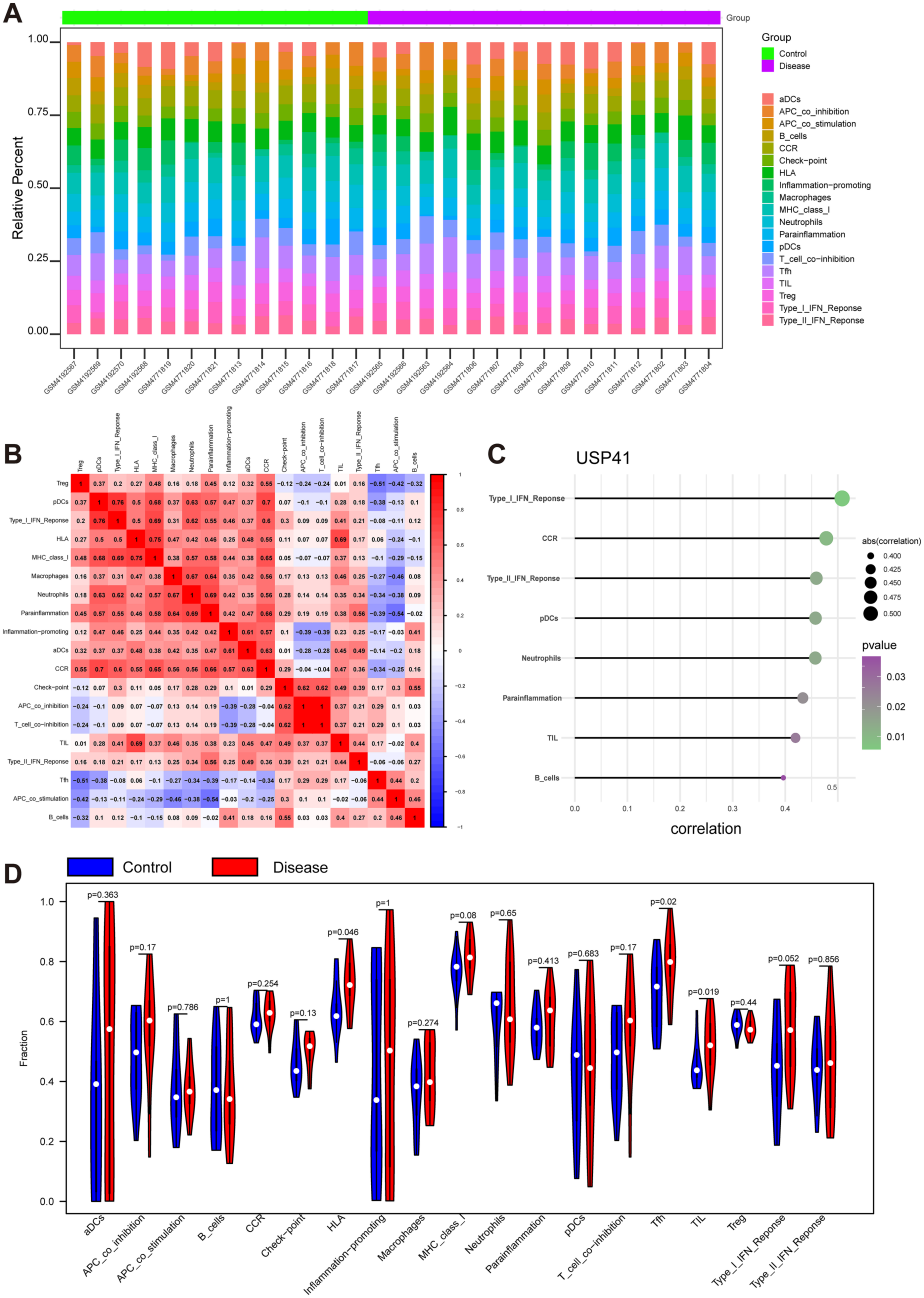
Analysis of infiltrating immune cells and immune-related pathways. **(A)** A heatmap visualizes the immune characteristic profiles between the Moyamoya disease (MMD) group and the control group. Each column stands for a sample, and each row represents an immune factor. **(B)** A correlation matrix depicts all immune cells. Immune cells with positive associations are colored red, while those with negative associations are colored blue. The significance threshold was set at *p* < 0.05. **(C)** The illustration shows that USP41 was significantly positively associated with type I IFN response, CCR, type II IFN response, plasmacytoid dendritic cells (pDCs), neutrophils, parainflammation, TILs, and B cells. The plot color represents the *p* value, and the plot size represents the correlation. **(D)** The violin plot shows the difference in all immune cells between the MMD and control groups.

To gain insights into the potential molecular mechanisms driving MMD progression, we conducted an in-depth exploration of the relationship between key genes and immune cell infiltration. By employing the ssGSEA algorithm, we assessed the immune cells present in the expression profiles and deduced the respective percentages of immune-infiltrating cells. The results showed the percentages of various immune cells in MMD and the correlation between the different kinds of immune cells (Figures 4A,B). These findings also suggested that the levels of human leukocyte antigen (HLA), follicular helper T cells (Tfh), and tumor-infiltrating lymphocytes (TIL) were significantly higher in patients with MMD than in controls (Figure 4D). The correlation analysis between key genes and immune cells revealed that ANAPC11 was significantly negatively associated with HLA, macrophages, and major histocompatibility complex (MHC) class I (Supplementary Figure S2F). UCHL1 exhibited a significant negative correlation with the type I interferon (IFN) response and HLA (Supplementary Figure S2F), whereas USP41 demonstrated a significant positive correlation with the type I IFN response, chemokine receptor (CCR), type II IFN response, plasmacytoid dendritic cells (pDCs), neutrophils, parainflammation, TILs and B cells (Figure 4C).

Additionally, we employed the TISIDB database (http://cis.hku.hk/TISIDB/) to explore the relationships between the three key genes and diverse immune elements. Our findings revealed that these key genes were intimately linked to the extent of immune cell infiltration and potentially held a crucial position within the immune microenvironment (Supplementary Figures S1A–E).

### Gene set enrichment analyses of key genes

The GSEA-based enrichment analysis illustrated that the pathways enriched by UCHL1 included base excision repair, Hippo signaling pathway, and Th1 and Th2 cell differentiation (Figures 2E,F). Pathways enriched by ANAPC11 included neutrophil extracellular trap formation, olfactory transduction, and oxidative phosphorylation (Figure 2G). Pathways enriched by UPS41 included the chemokine signaling pathway, NF-kappa B signaling pathway, and phagosome (Figure 2H).

### Prediction of regulatory networks of key genes

Taking the three key genes (*ANAPC11, UCHL1,* and *USP41*) as the gene set, we predicted the associated transcription factors using the Cistrome database and constructed transcriptional regulatory networks. A total of 76 transcriptional regulators were predicted by ANAPC11, 97 by UCHL1, and 99 by USP41 (Supplementary Figure S3). Employing the Mircode database for the purpose of anticipating the expression patterns of these pivotal genes in reverse, we obtained 30 miRNAs and 40 messenger RNA (mRNA)-miRNA relationship pairs. The visualization of this results was also performed with Cytoscape software (Supplementary Figure S4).

### Analysis of secondary-related genes involved in MMD

**Figure 5:**
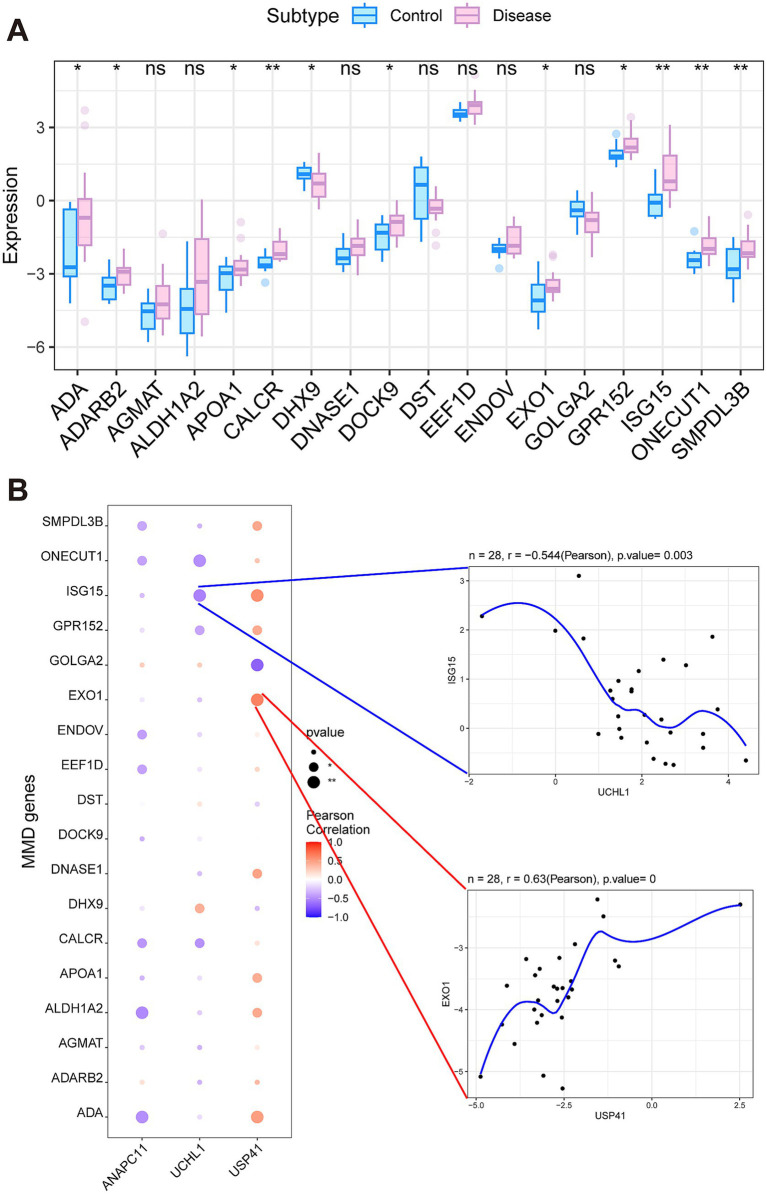
Analysis of secondary-related genes involved in Moyamoya disease. **(A)** The significantly differentially expressed genes between the MMD and control groups. **(B)** The correlation between the key genes and the secondary-related genes.

We retrieved MMD-associated genes from the GeneCards database (https://www.genecards.org/), and selected those genes that exhibited the highest correlation scores related to MMD. The expression differences of these MMD-related genes between MMD patients and controls were analyzed. We found that the expression levels of ADA, ADARB2, APOA1, CALCR, DHX9, DOCK9, EXO1, GPR152, ISG15, ONECUT1, and SMPDL3B indicated notable variations between the two groups (Figure 5A). An analysis of the correlation between three crucial genes and a selection of MMD-associated genes indicated that USP41 and EXO1 exhibited a notable positive correlation (Pearson’ s *r* = 0.63), whereas UCHL1 and ISG15 displayed a significant negative correlation (Pearson’ s *r* = −0.544) (Figure 5B).

### Prediction of potential drugs

The top 150 differentially upregulated and downregulated genes were divided into two groups, and the CMap database was utilized for drug prediction. The results suggested that the expression profiles of drug perturbations such as benzohydroxamic-acid, PKC-beta Inhibitor, quinoclamine, and XMD-1150 were more significantly negatively correlated with the expression profiles of MMD perturbations. These findings suggest that these drugs might be able to attenuate or even reverse the state of MMD.

## Discussion

MMD is a rare and unique cerebrovascular condition, first documented in 1969. It is distinguished by stenosis or occlusion in the terminal segment of the internal carotid artery, which results in the development of an aberrant vascular network comprising collateral pathways at the base of the brain. Reducing its incidence and prevalence is crucial for improving patient outcomes (17). For decades, a considerable number of neuroscientists have worked on MMD. However, its pathology remains unclear.

The pathological studies of the abnormal intracranial vessels in MMD have revealed a series of pathological changes, including the thickening of the intima due to fibrocellular proliferation, accompanied by an elevated count of smooth muscle cells, and conspicuous waviness in the internal elastic lamina, along with a thinning of the media (18). However, the mechanisms underlying these abnormal pathological findings are largely unknown. An epidemiological study reported that approximately 15% of the patients of MMD in Japan are familial cases, and pedigree analysis suggested that this disease might be autosomal dominant with incomplete penetrance (19). Although its pathogenic genes have not yet been identified, multiple studies have suggested that MMD was associated with specific genetic factors (20). In 2011, the specific variations leading to SNPs (preponderantly p. R4810K and p. R4859K) in *RNF213*, which were associated with the familial and sporadic cases in Japanese, Korean, and Chinese individuals, were independently identified by two research groups (8, 9). Subsequently, a number of additional RNF213 variants were detected in East Asian and Caucasian patients who did not have the p. R4810K mutation (8, 10, 11). *RNF213* is regarded as a gene that predisposes individuals to MMD, and the importance of MMD’s genetic background is receiving growing attention. RNF213, a cytosolic protein with a molecular weight of 591-kDa, is composed of two distinct functional domains, which exhibit six ATPase units and a multidomain E3 ubiquitin ligases module, and the pathologic mutations in MMD cluster in the E3 domain which not only result in a decline in the ubiquitin ligase activity of RNF213 but also boost the activation of NF-kappa B and apoptosis in an AAA + domain-dependent manner (8, 12, 13). Additionally, researchers have characterized the ubiquitin ligase function of RNF213 *in vitro* and discovered that mutations in RNF213 associated with MMD can result in a substantial decrease in its ubiquitin ligase activity, which suggests that diminished ubiquitin ligase activity, caused by mutations in RNF213, may contribute to the development of MMD (13). Therefore, ubiquitination may be an important biological process that contributes to MMD.

In our study, we selected and downloaded the GSE157628 and GSE141024 datasets related to MMD from the GEO database, including expression profiling data from 28 patients. Then 1,632 DEGs were identified by screening for differential expression between the MMD and control groups. Among them, 794 were upregulated and 838 were downregulated. Next, we extracted ubiquitination-related genes with relevance scores > 10 using the GeneCards database and took the intersection of these genes and the DEGs described above. We identified 25 intersecting genes that were ubiquitination-related and differentially expressed. Subsequently, LASSO regression and the SVM algorithm were employed to further select the features of these intersecting genes and identified seven and eight feature genes, respectively. Finally, we examined the intersection of these genes and obtained three key genes for our study, ANAPC11, UCHL1, and USP41.

Furthermore, we measured the blood samples of MMD patients and normal controls, and the results showed that the serum UCHL1 content in MMD group was significantly lower than that in HC group. This is consistent with the findings of bioinformatics mentioned above. In addition, we further conducted cell scratch experiments, and the results showed that compared with the negative control group, the migration ability of HBVSMC cells in the UCHL1 knockout group was significantly improved. These results suggest that the decreased expression level of UCHL1 may affect the pathogenesis of MMD by promoting the migration of vascular smooth muscle cells and other mechanisms. And the significant decrease in UCHL1 expression levels in the peripheral blood of MMD patients that we discovered provides innovative theoretical basis for the development of clinical diagnostic tools, such as test kits and rapid test strips. Further research is needed to explore its mechanism and application in MMD.

*UCHL1* gene encodes the ubiquitin carboxyl-terminal hydrolase L1 (UCHL1), which is a critical a member of the deubiquitinating enzyme family for removing ubiquitin or polyubiquitin from target proteins (21, 22). UCHL1, a protein of diverse functions, is capable of cleaving free monoubiquitin from ubiquitinated proteins, thus facilitating its reuse, conjugating ubiquitin to certain proteins, and binding to free monoubiquitin, ensuring the maintenance of an adequate supply of available ubiquitin (23, 24). Human UCHL1 was first discovered in the brain and other organs using two-dimensional electrophoresis and was termed protein gene product 9.5 (PGP9.5) (25). Later, ubiquitin C-terminal hydrolase activity was discovered (23). UCHL1 is neuron-specific and one of the most abundantly expressed proteins in the brain, accounting for approximately 1–5% of the soluble proteins, with a minor proportion tightly bound to membranes in the brain (26, 27). UCHL1 is essential for maintaining ubiquitin homeostasis. It may also be important in regulating other neuronal processes, in addition to the ubiquitin-proteasome pathway. Under pathological conditions, unfolded UCHL1 may also inhibit autophagy (28, 29). UCHL1 may also be related to the neuronal cytoskeleton proteins and may be involved in the regulation of axonal transport and maintenance of axonal integrity (28, 30). Mutations or deletions in UCHL1 result in axonal and dendritic pathologies, particularly affecting the motor systems (31–33). It might also be involved in memory function by regulating the function of synapses under some conditions (34). Mutations and functional aberrations of UCHL1 are associated with several neurological disorders. Several neurodegenerative diseases, including Parkinson’s disease, Alzheimer’s disease, and amyotrophic lateral sclerosis, have been linked to UCHL1 dysfunction (35). The injury mechanisms and the processes of recovery after suffering from traumatic brain injury and cerebral ischemia are both closely related to this dysfunction (36). Cerebral ischemia leads to the forming of various reactive lipids and other molecules, such as cyclopentenone prostaglandins (CyPGs) and nitric oxide (NO) (36–38). The cysteine at cysteine 152 (C152) of UCHL1 could be covalently modified by CyPGs and NO, which inhibits its activity, unfold the enzyme, and lead to protein aggregation (36–40). Multiple studies have indicated that UCHL1 could play a part in determining the survival of gray and white matter, alleviating gray and white matter damage, and contributing to motor recovery post-cerebral ischemia (36, 40–44). Besides, an article on ischemic heart injury reported that UCHL1 might play a novel protective role on myocardial infarction via stabilizing hypoxia-inducible factor 1 alpha and promoting its signaling (45). This suggests that there may be other undiscovered pathways through which UCHL1 affects neurological disorders, such as cerebral ischemia. Therefore, further research on UCHL1 expression is warranted.

Studies on ANAPC11 and USP41 are limited. The ANAPC11 gene is widely spread across the cytoplasm and nucleus, and discrete aggregates can be observed in the granular structures (46). In northern blot hybridization, the signal intensity varied throughout the body and was higher in organs such as skeletal muscle, heart, brain and kidneys (46). ANAPC11 has notably high expression in particular cancer types and displays distinct expression profiles across different cancer cell lines (47). In contrast to the expression levels in normal tissues, ANAPC11 is overexpressed in leukemia and lung cancer cell lines (46). USP41 is overexpressed in lung cancer tissues, osteosarcoma cell lines, and breast cancer (48). These results suggest that the pathomechanism of MMD is complex and may be associated with certain tumor-promoting factors, which require further research.

In addition to gene factors, microenvironmental factors also play a role in MMD pathogenesis (3, 4). Immune factors are extensively involved in the composition of the microenvironment. Due to the absence of pathological evidence, it is not clear if inflammatory and immune factors have an impact on the pathogenesis of MMD. Nevertheless, an immunohistochemical examination of autopsy specimens from MMD cases disclosed that the expression of immunoglobulin G in the internal elastic lamina of the internal carotid artery (ICA) and middle cerebral artery deviated from normal levels, which implies that the autoimmune response may play a role in the pathogenesis of MMD (49). In addition, a high-density autoantibody array revealed that the levels of 165 autoantibodies in the serum of MMD patients were elevated relative to the control group, and six of these autoantibodies were identified as being specific to MMD (50). Recently, bioinformatics studies have reported the involvement of immune cell infiltration and activity in MMD progression (14, 15). Nevertheless, additional research is essential to clarify the role of autoimmunity in the progression of MMD. To delve deeper into the potential molecular mechanisms underlying MMD progression, we analyzed the connection between the key genes identified above and immune infiltration. Initially, we utilized the ssGSEA algorithm to quantify the immune cells within the expression profiles, aiming to infer the relative proportions of 29 types of immune-infiltrating cells. The results indicated that the levels of HLA, Tfh, and TIL in the MMD group were notably higher than those in the control group. We then employed Spearman’s rank correlation coefficient to explore the correlations between the three key genes and immune cells. The results showed that ANAPC11 had a significant negative correlation with HLA, macrophages, and MHC class I; UCHL1 exhibited a significant negative correlation with the type I IFN response and HLA, whereas USP41 demonstrated a significant positive correlation with the type I IFN response, CCR, type II IFN response, plasmacytoid dendritic cells (pDCs), neutrophils, inflammation, TILs, and B cells.

Moreover, we employed the TISIDB database to explore the relationships between the three key genes and diverse immune elements, such as immunostimulatory factors, immunosuppressive factors, chemokines, and receptors. Our findings revealed that these key genes were intimately linked to the extent of immune cell infiltration and potentially held a crucial position within the immune microenvironment.

Furthermore, we conducted an in-depth enrichment analysis focusing on the three key genes associated with MMD. The results demonstrated that the pathways enriched by ANAPC11 encompassed neutrophil extracellular trap formation, olfactory transduction, and oxidative phosphorylation. For UCHL1, the enriched pathways involved base excision repair, the Hippo signaling pathway, as well as Th1 and Th2 cell differentiation. Regarding USP41, the chemokine signaling pathway, the NF-kappa B signaling pathway, and the phagosome were among the enriched pathways. Neutrophil extracellular traps (NETs) are DNA structures decorated with cytosolic, granular, and nuclear proteins that can entrap microorganism, and the release of NETs might be influenced by unbalanced immune responses, leading to a variety of disorders (51). Mechanisms underlying NET formation are complex (52). There are several known mechanisms for NET formation, and this process varies in different physiological environments, including blood and tissues, and under alkaline or hypertonic conditions (52). NETs could induce a wide range of pathological processes and plays a role in autoimmune immunodeficiencies, diabetes and cardiovascular diseases, tumors and cystic fibrosis (51). One of the pathogenic functions of NET is occlusion, in which NETs frequently converge in intravascular thrombi and occluded conduits, blocking the circulation and secretion of blood and other fluids (53). Base excision repair can correct oxidative, deamination, alkylation, and basic single-base damage. Base excision repair (BER) genes deficiency contributes to cancer, inflammation, aging, and neurodegenerative disorders (54). The Hippo pathway has now been implicated in a variety of human diseases such as cancer, autoimmunity and so on (55). The transcription factor family of NF-kappa B serves as a crucial stress-responsive element within the cellular milieu, and it exerts a regulatory function over the expression of key genes related to multiple biological processes, such as immunity, inflammation, cell death, and proliferation (56). Studies have shown that RNF213 can also enhance NF-kappa B activation, which might be part of the pathological process of MMD (13). Thus, the outcomes of these pathway analyses further indicate that the three key genes may play critical regulatory roles in the pathogenesis of MMD. However, the specific mechanisms of these pathways in MMD remain unclear. And incorporating more experimental validation can be further explored to enhance the persuasiveness of the conclusions. Our team will continue to investigate the potential pathways of ubiquitination in MMD in future studies.

We predicted the key gene-associated transcription factors and constructed transcriptional regulatory networks using the Cistrome database. A total of 76 transcriptional regulators were predicted by ANAPC11, 97 by UCHL1, and 99 by USP41. The appearance of proteins during MMD progression were modified by gene-miRNAs by targeting their primary targets. In our research, we built a gene-miRNA regulatory network. Employing the Mircode database for the purpose of anticipating the expression patterns of these pivotal genes in reverse, we obtained 30 miRNAs and 40 mRNA-miRNA relationship pairs. These miRNAs can be targeted for intervention in the study of MMD pathogenesis.

We retrieved MMD-related genes from the GeneCards database. We selected the genes with the highest relevance scores and conducted an analysis of the expression variations between the MMD group and the control group to reduce redundancy. The results indicated that there were significant differences in the expression levels of ADA, ADARB2, APOA1, CALCR, DHX9, DOCK9, EXO1, GPR152, ISG15, ONECUT1, and SMPDL3B between the two groups. Moreover, we examined the correlations between the expression levels of the three key genes and those of several selected genes. It turned out that USP41 and EXO1 were significantly positively correlated (Pearson’s *r* = 0.63), while UCHL1 and ISG15 were significantly negatively correlated (Pearson *r* = −0.544). ISG15, an interferon-stimulated ubiquitin-like protein, attaches to substrate proteins through a process known as ISGylation, playing a role in the body’s defense against microbial infections (57). Interferon is capable of triggering the ISGylation and oligomerization of RNF213 on lipid droplets, during which RNF213 acts as a detector for ISGylated proteins, suggesting that it serves as a vital antimicrobial effector (57). Further research is essential to clarify the roles these genes play in the development of MMD.

In addition, our study identified 13 potential medications or chemical entities as treatment options for MMD by focusing on key ARGs. The top 150 genes with differentially upregulated and downregulated expressions were grouped into two categories, and the Connectivity Map database was utilized for drug prediction. The outcomes indicated that the expression profiles of drug interferences like benzohydroxamic-acid, PKC-beta inhibitor, quinoclamine, and XMD-1150 were more remarkably negatively correlated with the expression profiles of MMD-related interferences. Previous observations suggest that benzohydroxamic-acid may inhibit tumor cell proliferation and metastasis through various mechanisms (58). The results suggest benzohydroxamic-acid may inhibit the proliferation and migration of vascular smooth muscle cells in MMD. Research data indicates that PKC-beta inhibitors help reduce the risk of severe cardiac microvascular ischemia/reperfusion injury in diabetic rats by maintaining endothelial barrier function and exerting anti-apoptotic effects (59). It may also play an endothelial protective role in MMD. The XMD-1150 compound has the potential to target one or multiple autophagy hub genes, thereby expediting the modulation of autophagy in the context of cancer therapy (60). By targeting one or multiple autophagy hub genes, XMD1150 could expedite the modulation of autophagy, a process crucial for cellular homeostasis and clearance of damaged or unnecessary cellular components. In the context of moyamoya disease, which involves abnormal blood vessel formation and ischemia, autophagy modulation might help to regulate vessel growth, reduce inflammation, or enhance the clearance of debris, thereby contributing to therapeutic benefits. These results imply that these drugs may be capable of alleviating or even reversing the condition of MMD. However, further research is needed to fully elucidate the specific pathways and mechanisms through which these drugs exert its effects in MMD treatment.

In summary, the expression level of UCHL1 gene is significantly reduced in MMD, which may affect the pathogenesis of MMD by promoting the migration of vascular smooth muscle cells. ANAPC11, UCHL1, and USP41 ubiquitination related genes may be significantly associated with the pathogenesis of MMD. In depth research on this correlation, *in vitro* and *in vivo* validation of gene miRNA regulatory networks, exploration of potential therapeutic drugs for MMD, and validation of drug effects are important directions for future research. There is a significant correlation between ubiquitination and the pathogenesis of MMD. The in-depth study of this correlation, the in vitro and in vivo validation of the gene-miRNA regulatory network, the exploration of potential therapeutic drugs for MMD, and the validation of drug effects are important directions for future research.

This study has several limitations. First, although we used a variety of methods in the feature selection process and verified the stability of the model through cross-validation, the sample size of this study still could lead to overfitting which might limit the reliability of the study model and the reliability of the identified ubiquitin-related genes. We also plan to expand the sample size in future studies, particularly by including blood and surgical specimens from children and adult MMD patients with fewer comorbidities, to conduct an in-depth investigation of ubiquitination-related genes in MMD. Secondly, in the selected datasets, control samples were obtained from patients with other diseases. Given the potential genetic and microenvironmental impacts of other diseases, the data from the control group might diverge from that of normal samples at the transcriptional level. Harvesting normal blood vessels from healthy controls is essential for ethical reasons. Therefore, it is acceptable to use samples from patients with other diseases as controls when studying MMD. Third, the results were based on bioinformatics and *in vitro* experiments which only verified the effect of UCHL1 on smooth muscle migration. Further animal experiments are crucial for verifying the role of ubiquitination modification in the pathogenesis of MMD. However, there are currently no animal models for MMD, and our findings provide a new potential theoretical basis for the development of an MMD animal model. Our team will continue to invest in research aimed at establishing a suitable animal model for MMD.

## Conclusion

In our study, these three ubiquitination-related genes, namely ANAPC11, UCHL1, and USP41, were identified as the key genes that might be involved in the pathogenesis of MMD, and these genes were closely associated with multiple signaling pathways of protein modification, autoimmune, immune response, neutrophil extracellular trap formation, base excision repair, Hippo signaling pathway, and NF − kappa B signaling pathway. In addition, we verified that the level of serum UCHL1 expression in MMD disease was significantly reduced by external data and in vitro experiments, and that UCHL1 gene knockout could promote the migration of vascular smooth muscle cells. These results suggest that UCHL1 may affect the migration ability of vascular smooth muscle cells through mechanisms such as ubiquitination modification, and thus participate in the pathogenesis of MMD. Using these three key genes, we identified two secondary genes: EXO1 and ISG15. Furthermore, we constructed a gene-miRNA network containing 30 miRNAs. Finally, we predicted that drugs targeting key genes might be effective in treating MMD.

## Data Availability

The original contributions presented in the study are included in the article/Supplementary material, further inquiries can be directed to the corresponding author/s.
