# Supplementary material for: Integrating machine learning for the identification of ubiquitination-associated genes in moyamoya disease
Source: Front Neurol. 2025 Sep 16;16:1653433. doi: 10.3389/fneur.2025.1653433 (PMC12479308; doi:10.3389/fneur.2025.1653433)

## Supplementary Information

Table S1 The GSEA results of ANAPC11.

| Description | enrichment Score | NES | pvalue | p.adjust | qvalue |
|---|---|---|---|---|---|
| Oxidative phosphorylation | 0.389070189 | 1.824751994 | 1.91E-05 | 0.003225032 | 0.002830654 |
| Cardiac muscle contraction | 0.388744464 | 1.720089642 | 0.000389275 | 0.032796453 | 0.028785892 |
| Olfactory transduction | 0.302768571 | 1.651563946 | 1.56E-08 | 5.24E-06 | 4.60E-06 |
| Herpes simplex virus 1 infection | -0.33644479 | -1.486402232 | 9.61E-05 | 0.010799572 | 0.00947893 |
| Neutrophil extracellular trap formation | -0.430845666 | -1.672482223 | 0.000722633 | 0.048705434 | 0.04274942 |

Table S2 The GSEA results of UCHL1.

| Description | enrichment Score | NES | pvalue | p.adjust | qvalue |
|---|---|---|---|---|---|
| Prostate cancer | 0.392255063 | 1.644807326 | 0.00185668 | 0.048130871 | 0.043297488 |
| Hippo signaling pathway | 0.350581161 | 1.580777681 | 0.001301641 | 0.039877556 | 0.035872985 |
| Coronavirus disease - COVID-19 | -0.345601974 | -1.574795537 | 0.000315213 | 0.02192165 | 0.019720241 |
| Herpes simplex virus 1 infection | -0.373245638 | -1.830079383 | 3.26E-10 | 1.10E-07 | 9.88E-08 |
| Th1 and Th2 cell differentiation | -0.418626701 | -1.674836768 | 0.001250186 | 0.039877556 | 0.035872985 |
| Staphylococcus aureus infection | -0.438409527 | -1.745238546 | 0.000389005 | 0.02192165 | 0.019720241 |
| Viral myocarditis | -0.478188078 | -1.782906647 | 0.000580479 | 0.024452688 | 0.021997108 |
| Base excision repair | -0.512541104 | -1.796975442 | 0.001637144 | 0.045976463 | 0.041359429 |
| Autoimmune thyroid disease | -0.530940123 | -1.906753686 | 0.000455346 | 0.02192165 | 0.019720241 |
| Allograft rejection | -0.576501696 | -1.970909412 | 0.000436212 | 0.02192165 | 0.019720241 |
| Asthma | -0.592662893 | -1.916427945 | 0.00079888 | 0.029913605 | 0.02690963 |
| Intestinal immune network for IgA production | -0.617328154 | -2.200996657 | 3.26E-06 | 0.000366508 | 0.000329702 |
| Systemic lupus erythematosus | -0.622658833 | -2.261304431 | 5.46E-07 | 9.21E-05 | 8.28E-05 |

Table S3 The GSEA results of UPS41.

| Description | enrichment Score | NES | pvalue | p.adjust | qvalue |
|---|---|---|---|---|---|
| Asthma | 0.736366952 | 2.473633639 | 8.45E-09 | 1.78E-07 | 1.25E-07 |
| Leishmaniasis | 0.696343466 | 2.876859648 | 1.00E-10 | 3.37E-09 | 2.37E-09 |
| Graft-versus-host disease | 0.692956696 | 2.495393983 | 3.42E-09 | 8.23E-08 | 5.78E-08 |
| Allograft rejection | 0.685799281 | 2.427262476 | 4.13E-08 | 6.96E-07 | 4.89E-07 |
| Type I diabetes mellitus | 0.66917512 | 2.441474064 | 9.20E-09 | 1.78E-07 | 1.25E-07 |
| Viral myocarditis | 0.664998726 | 2.592688204 | 1.00E-10 | 3.37E-09 | 2.37E-09 |
| Intestinal immune network for IgA production | 0.663468566 | 2.483576498 | 1.84E-09 | 4.76E-08 | 3.35E-08 |
| Systemic lupus erythematosus | 0.655168405 | 2.462490842 | 8.24E-10 | 2.52E-08 | 1.77E-08 |
| Staphylococcus aureus infection | 0.631998433 | 2.700871865 | 1.00E-10 | 3.37E-09 | 2.37E-09 |
| Viral protein interaction with cytokine and cytokine receptor | 0.609729153 | 2.608396759 | 1.00E-10 | 3.37E-09 | 2.37E-09 |
| Hematopoietic cell lineage | 0.60548284 | 2.595066529 | 1.00E-10 | 3.37E-09 | 2.37E-09 |
| Autoimmune thyroid disease | 0.590656393 | 2.225245509 | 2.09E-06 | 2.43E-05 | 1.71E-05 |
| Inflammatory bowel disease | 0.58969876 | 2.347439554 | 1.11E-08 | 1.97E-07 | 1.38E-07 |
| Antigen processing and presentation | 0.576810492 | 2.353705008 | 9.52E-09 | 1.78E-07 | 1.25E-07 |
| Malaria | 0.566992693 | 2.110223036 | 9.01E-06 | 9.20E-05 | 6.47E-05 |
| Neutrophil extracellular trap formation | 0.547636525 | 2.380129464 | 1.00E-10 | 3.37E-09 | 2.37E-09 |
| Legionellosis | 0.546905631 | 2.095792579 | 7.37E-06 | 7.76E-05 | 5.45E-05 |
| Rheumatoid arthritis | 0.516290147 | 2.189672126 | 1.62E-07 | 2.49E-06 | 1.75E-06 |
| Phagosome | 0.506058574 | 2.322211491 | 1.00E-10 | 3.37E-09 | 2.37E-09 |
| Complement and coagulation | 0.49791776 | 2.110534 | 8.03E-07 | 1.08E-05 | 7.61E-06 |

| | | | | | |
|---|---|---|---|---|---|
| cascades | 5 | 04 | | | |
| Pertussis | 0.491188041 | 2.029284578 | 1.09E-05 | 0.0001076 | 7.56E-05 |
| Th1 and Th2 cell differentiation | 0.483279567 | 2.065528309 | 1.62E-06 | 1.95E-05 | 1.37E-05 |
| African trypanosomiasis | 0.483193023 | 1.684207419 | 0.006268037 | 0.031063654 | 0.021831398 |
| Tuberculosis | 0.479595186 | 2.249887142 | 1.00E-10 | 3.37E-09 | 2.37E-09 |
| Nicotine addiction | 0.478734608 | 1.723962649 | 0.002873712 | 0.016140684 | 0.011343601 |
| Toxoplasmosis | 0.467991394 | 2.055557675 | 9.91E-07 | 1.28E-05 | 9.03E-06 |
| Natural killer cell mediated cytotoxicity | 0.465977612 | 2.050951436 | 1.48E-07 | 2.37E-06 | 1.66E-06 |
| Cytokine-cytokine receptor interaction | 0.464102582 | 2.324935654 | 1.00E-10 | 3.37E-09 | 2.37E-09 |
| B cell receptor signaling pathway | 0.455758065 | 1.926395434 | 4.38E-05 | 0.000388035 | 0.00027271 |
| Osteoclast differentiation | 0.454808696 | 2.049701624 | 2.69E-07 | 3.93E-06 | 2.77E-06 |
| Fat digestion and absorption | 0.453263771 | 1.632240073 | 0.007035968 | 0.033396074 | 0.023470612 |
| Th17 cell differentiation | 0.446698233 | 1.944008365 | 5.73E-06 | 6.23E-05 | 4.38E-05 |
| NF-kappa B signaling pathway | 0.444684282 | 1.905892651 | 3.07E-05 | 0.000279497 | 0.000196429 |
| Chemokine signaling pathway | 0.434254795 | 2.065400236 | 5.75E-09 | 1.29E-07 | 9.09E-08 |
| Ovarian steroidogenesis | 0.429990557 | 1.613935968 | 0.00721939 | 0.033790758 | 0.023747995 |
| Cell adhesion molecules | 0.429584717 | 1.972793372 | 4.52E-07 | 6.35E-06 | 4.46E-06 |
| Mineral absorption | 0.425607682 | 1.659353579 | 0.003008768 | 0.016622208 | 0.011682014 |
| Fc gamma R-mediated phagocytosis | 0.41298712 | 1.764789375 | 0.000479501 | 0.003672543 | 0.002581047 |
| Fc epsilon RI signaling pathway | 0.412608186 | 1.648059725 | 0.004387581 | 0.022068875 | 0.015509908 |
| NOD-like receptor signaling pathway | 0.407873938 | 1.914861366 | 1.08E-06 | 1.35E-05 | 9.48E-06 |
| Amoebiasis | 0.404729358 | 1.746394605 | 0.000266388 | 0.00213745 | 0.00150219 |
| Neuroactive ligand-receptor | 0.40385372 | 2.071225 | 1.00E-10 | 3.37E-09 | 2.37E-09 |

| | | | | | |
|---|---|---|---|---|---|
| interaction | 2 | 506 | | | |
| Leukocyte transendothelial migration | 0.399866421 | 1.753264939 | 0.000151324 | 0.001243814 | 0.000874147 |
| Toll-like receptor signaling pathway | 0.381384745 | 1.634354923 | 0.00197429 | 0.012553502 | 0.008822545 |
| Influenza A | 0.377890267 | 1.762588228 | 2.92E-05 | 0.000273282 | 0.000192061 |
| Coronavirus disease - COVID-19 | 0.372320741 | 1.81320498 | 2.32E-06 | 2.60E-05 | 1.83E-05 |
| Epstein-Barr virus infection | 0.368912173 | 1.764009787 | 2.27E-05 | 0.000218756 | 0.000153741 |
| IL-17 signaling pathway | 0.367188167 | 1.569355722 | 0.006557593 | 0.032027662 | 0.022508899 |
| Olfactory transduction | 0.359223197 | 1.864036684 | 1.77E-09 | 4.76E-08 | 3.35E-08 |
| Measles | 0.352453446 | 1.583362736 | 0.001141778 | 0.007852633 | 0.005518796 |
| Yersinia infection | 0.351184502 | 1.582694991 | 0.001200577 | 0.007933223 | 0.005575434 |
| C-type lectin receptor signaling pathway | 0.349377663 | 1.517062786 | 0.007989979 | 0.03590164 | 0.025231513 |
| JAK-STAT signaling pathway | 0.338021583 | 1.560449634 | 0.001749159 | 0.011335893 | 0.007966816 |
| Lipid and atherosclerosis | 0.328235896 | 1.587921438 | 0.00064869 | 0.004857967 | 0.003414158 |
| Rap1 signaling pathway | 0.295363161 | 1.431033368 | 0.007753812 | 0.035794993 | 0.025156562 |
| Calcium signaling pathway | 0.276377952 | 1.362226123 | 0.009611277 | 0.042618428 | 0.029952042 |
| Herpes simplex virus 1 infection | -0.261213471 | -1.416624332 | 0.000840853 | 0.006029096 | 0.004237222 |
| Wnt signaling pathway | -0.319163759 | -1.536332536 | 0.00272166 | 0.015545754 | 0.010925487 |
| Ubiquitin mediated proteolysis | -0.330708933 | -1.543127476 | 0.003577394 | 0.018837213 | 0.01323871 |
| Autophagy - animal | -0.33349124 | -1.60003809 | 0.000868623 | 0.006098455 | 0.004285967 |
| Oxidative phosphorylation | -0.340923704 | -1.5461431 | 0.003991096 | 0.02037878 | 0.014322116 |
| TGF-beta signaling pathway | -0.355728628 | -1.573436757 | 0.003447424 | 0.018656666 | 0.013111822 |
| Ribosome biogenesis in eukaryotes | -0.357083427 | -1.451883093 | 0.010320334 | 0.045168213 | 0.03174402 |
| Thyroid hormone signaling | -0.3584874 | -1.631994 | 0.000809 | 0.005933 | 0.004170 |

| | | | | | |
|---|---|---|---|---|---|
| pathway | 54 | 861 | 937 | 67 | 157 |
| Peroxisome | -0.366851508 | -1.534466233 | 0.006752498 | 0.032508456 | 0.022846799 |
| ATP-dependent chromatin remodeling | -0.36779165 | -1.556567921 | 0.003778254 | 0.019588795 | 0.013766919 |
| mRNA surveillance pathway | -0.368708794 | -1.586969835 | 0.003487745 | 0.018656666 | 0.013111822 |
| RNA degradation | -0.388775746 | -1.580742453 | 0.002635253 | 0.015311731 | 0.010761017 |
| Pyruvate metabolism | -0.438483806 | -1.618724352 | 0.007971132 | 0.03590164 | 0.025231513 |
| Basal transcription factors | -0.476764469 | -1.743628191 | 0.002288667 | 0.013772871 | 0.009679512 |
| Fatty acid degradation | -0.484654984 | -1.780999506 | 0.002150444 | 0.013420361 | 0.00943177 |
| DNA replication | -0.490482305 | -1.738847242 | 0.002360919 | 0.013958413 | 0.009809911 |
| Nucleotide excision repair | -0.497374212 | -1.928934239 | 5.80E-05 | 0.000501463 | 0.000352426 |
| Glycosylphosphatidylinositol (GPI)-anchor biosynthesis | -0.517466155 | -1.69028225 | 0.011399458 | 0.049251505 | 0.034613739 |
| Valine | 47 | -0.524745175 | -1.959270228 | 0.000143605 | 0.001209869 |
| Circadian rhythm | -0.532559994 | -1.842167597 | 0.00224761 | 0.013771721 | 0.009678704 |
| Propanoate metabolism | -0.579673025 | -1.983513886 | 0.000315983 | 0.002476425 | 0.00174042 |
| Mismatch repair | -0.612449657 | -1.937207199 | 0.001188106 | 0.007933223 | 0.005575434 |

Figure S1 Principal component analysis before correction.

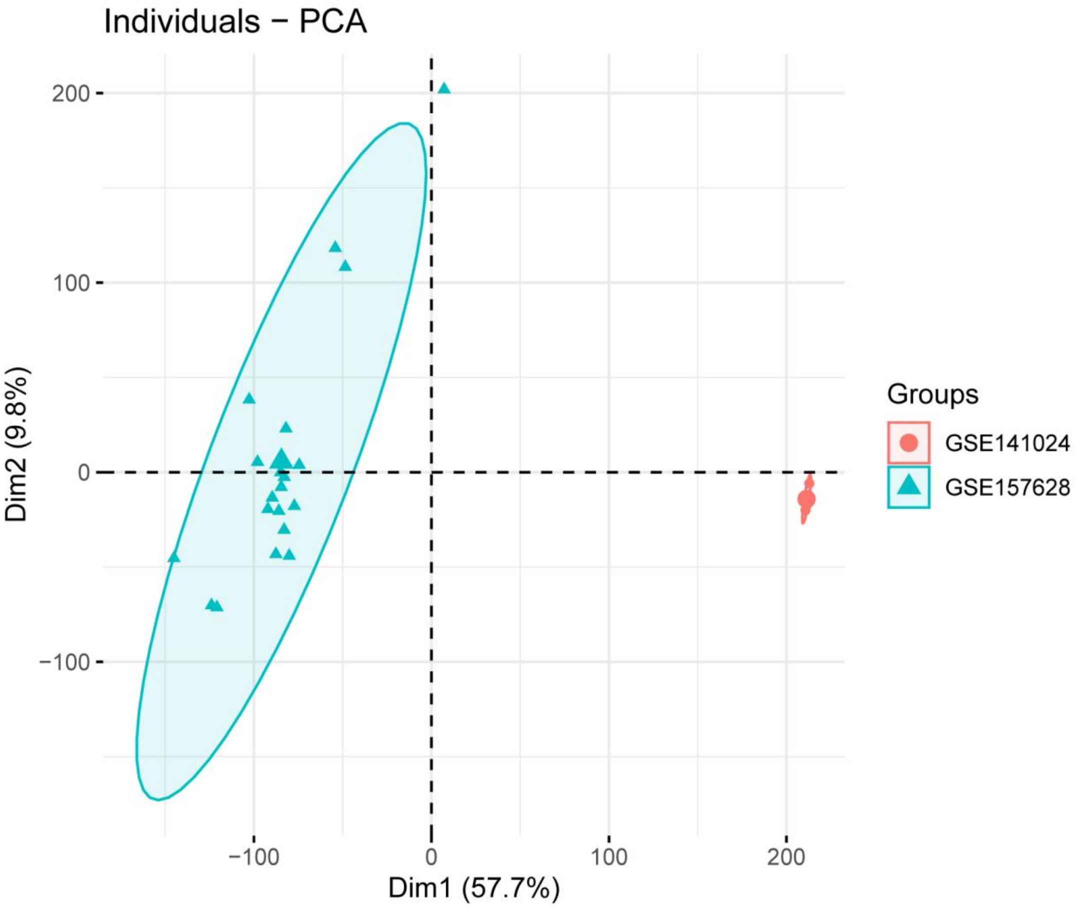

Figure S2 Analysis of infiltrating immune cells and immune-related pathways. (A), (B), (C), (D), and (E) The illustrations show the relationship between the three key genes and immune factors, including chemokine, immunoinhibitor, immunostimulator, major histocompatibility complex (MHC), and chemokine receptor. The plot color represents the correlation and the plot size represents the p value. (F) The illustration shows that ANAPC11 was significantly negatively associated with human leukocyte antigen (HLA), macrophages, MHC class I, and USP41 was significantly positively associated with type I interferon (IFN) response and HLA. The plot color represents the p value, and the plot size represents the correlation.

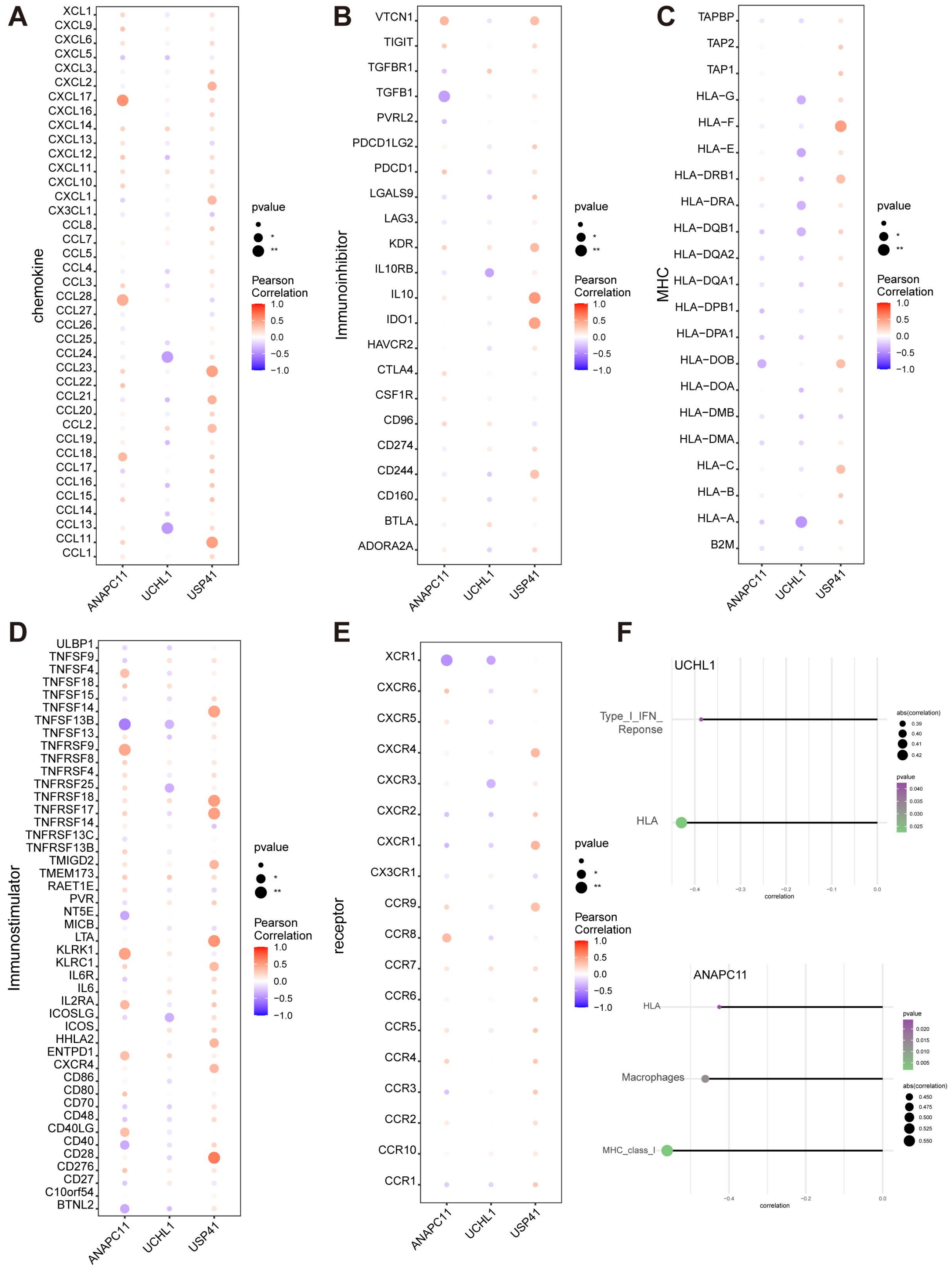

Figure S3 The transcription factor-gene regulatory network.

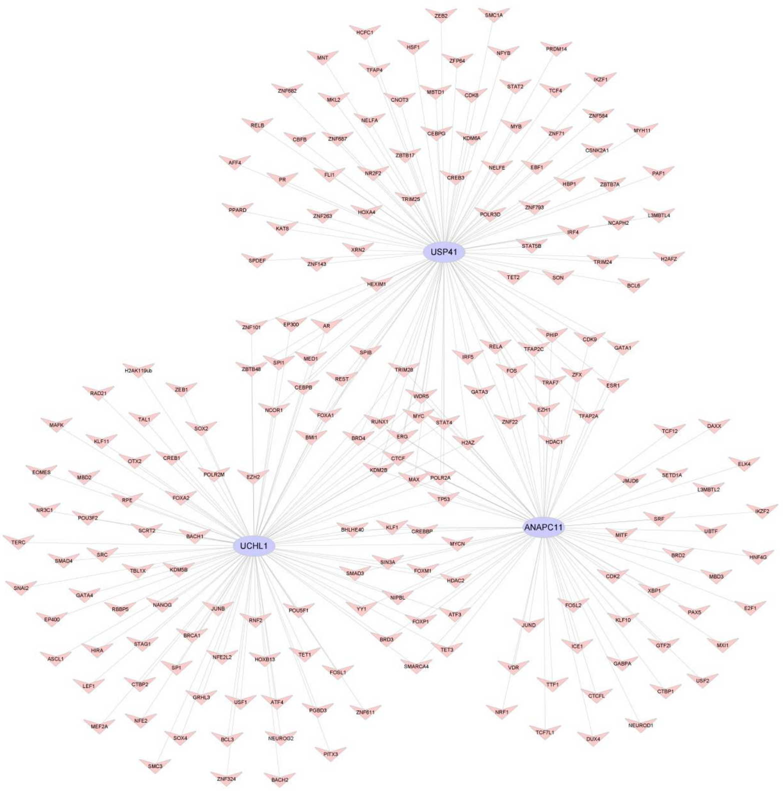

Figure S4 The miRNA-gene regulatory network.

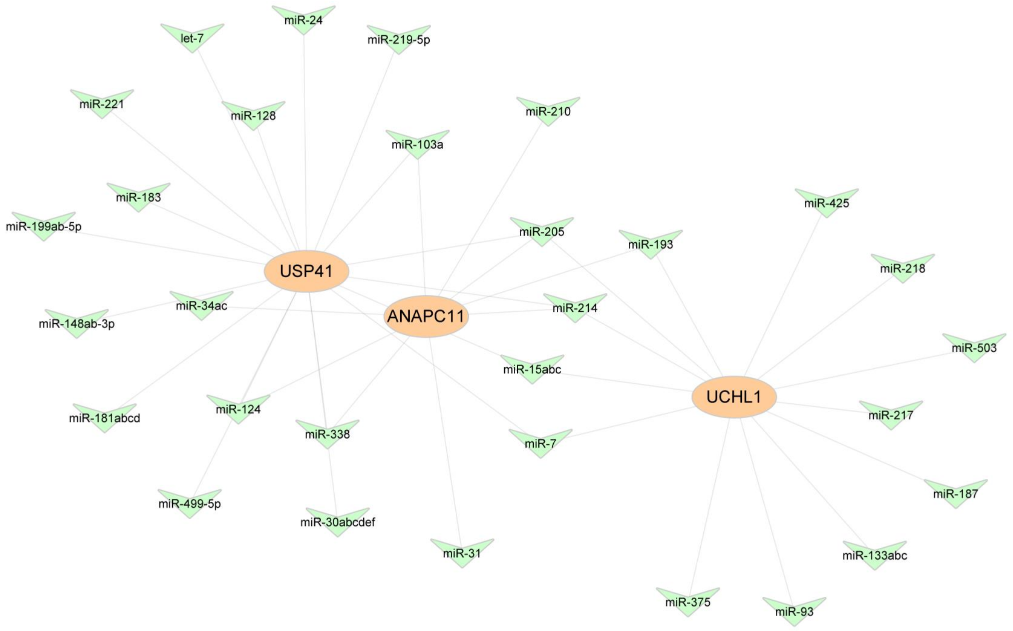

Supplement: Supplementary file 1 [file Data_Sheet_1.pdf]
