# Supplementary figures and images for: Integrating machine learning for the identification of ubiquitination-associated genes in moyamoya disease
Source: Front Neurol. 2025 Sep 16;16:1653433. doi: 10.3389/fneur.2025.1653433 (PMC12479308; doi:10.3389/fneur.2025.1653433)

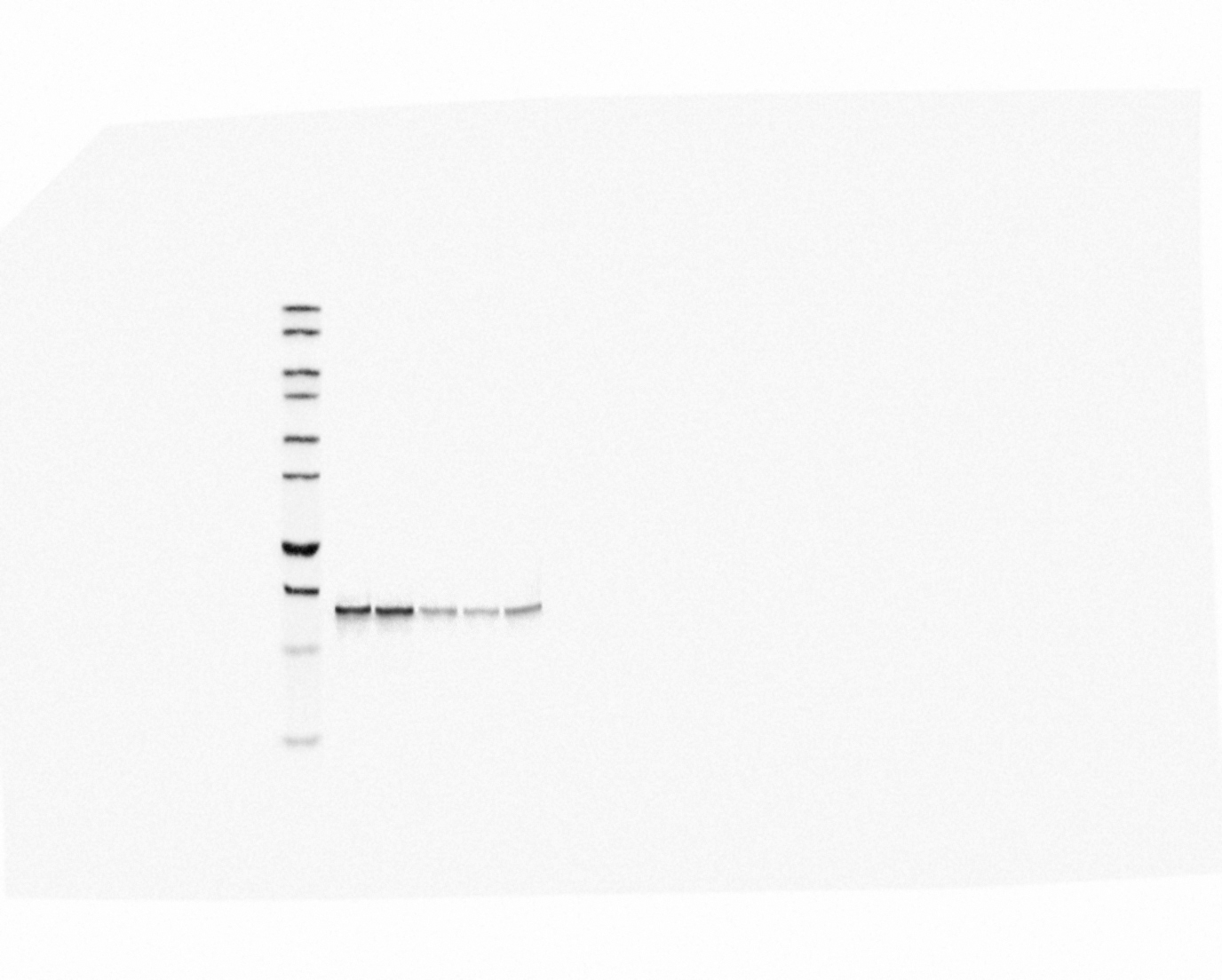

Supplement: Supplementary file 2 [file Data_Sheet_2.zip › Original Western Blot Images for Manuscript EPI-S2024-0015.R2/UCHL1-1.jpg]

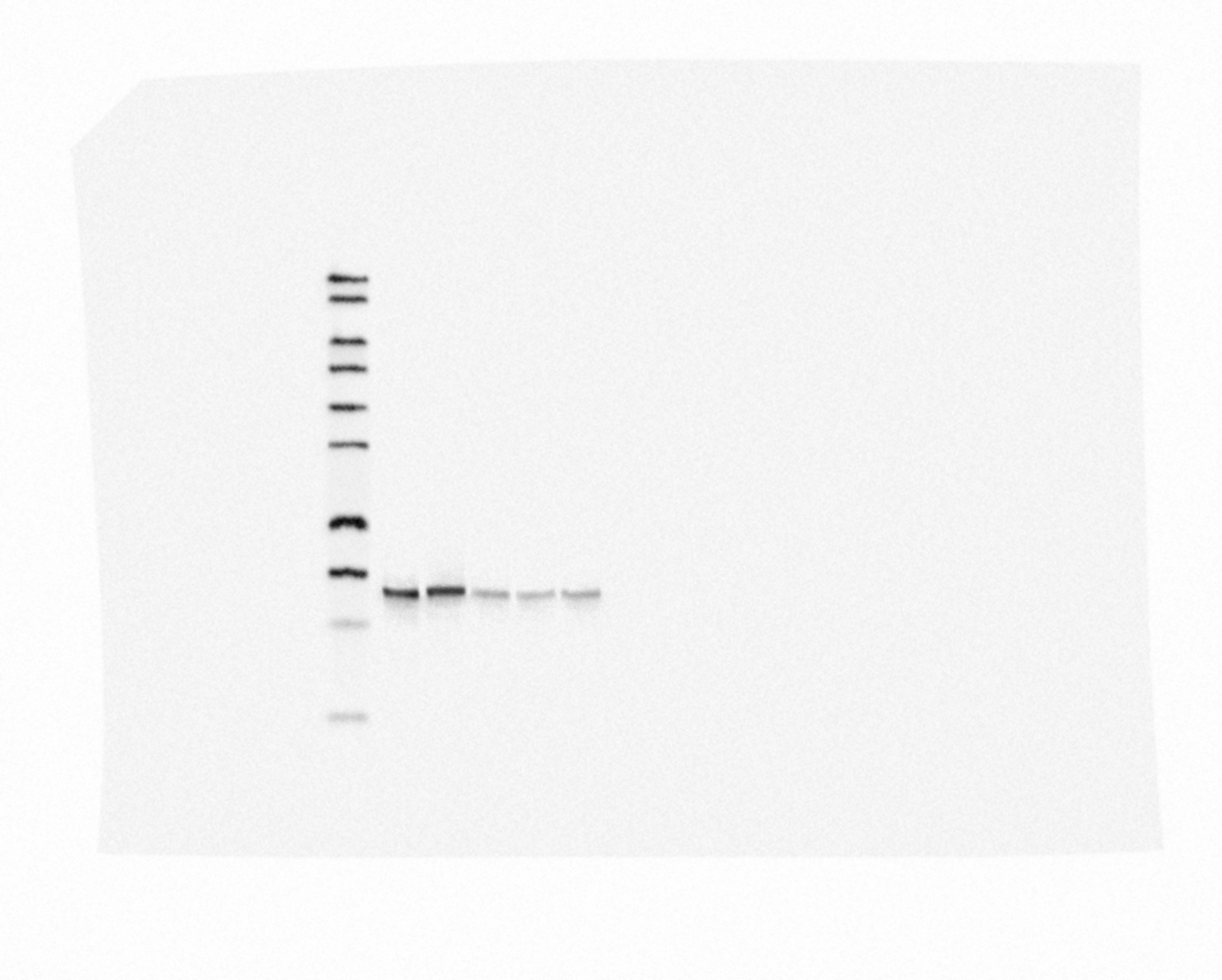

Supplement: Supplementary file 2 [file Data_Sheet_2.zip › Original Western Blot Images for Manuscript EPI-S2024-0015.R2/UCHL1-2.jpg]

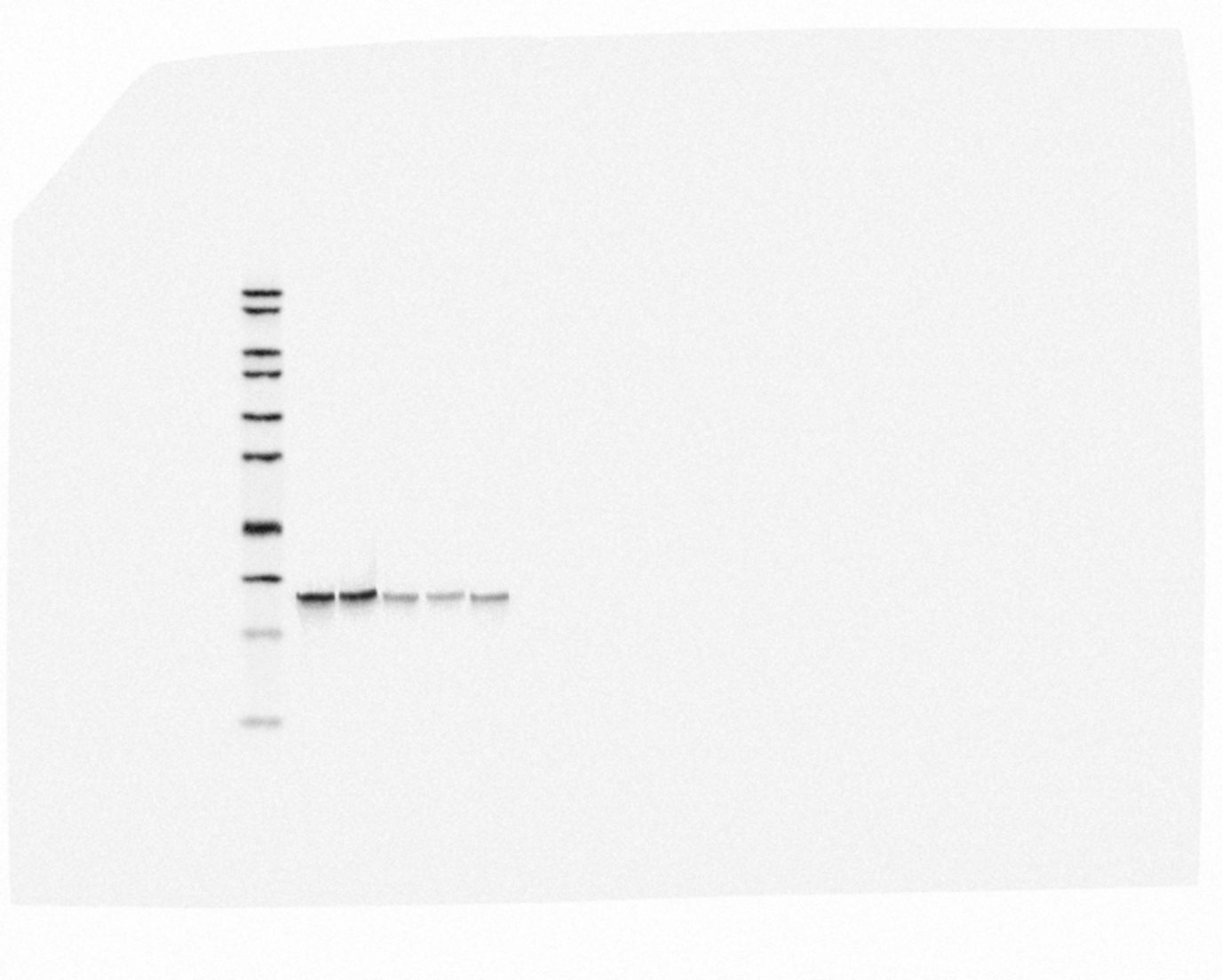

Supplement: Supplementary file 2 [file Data_Sheet_2.zip › Original Western Blot Images for Manuscript EPI-S2024-0015.R2/UCHL1-3.jpg]

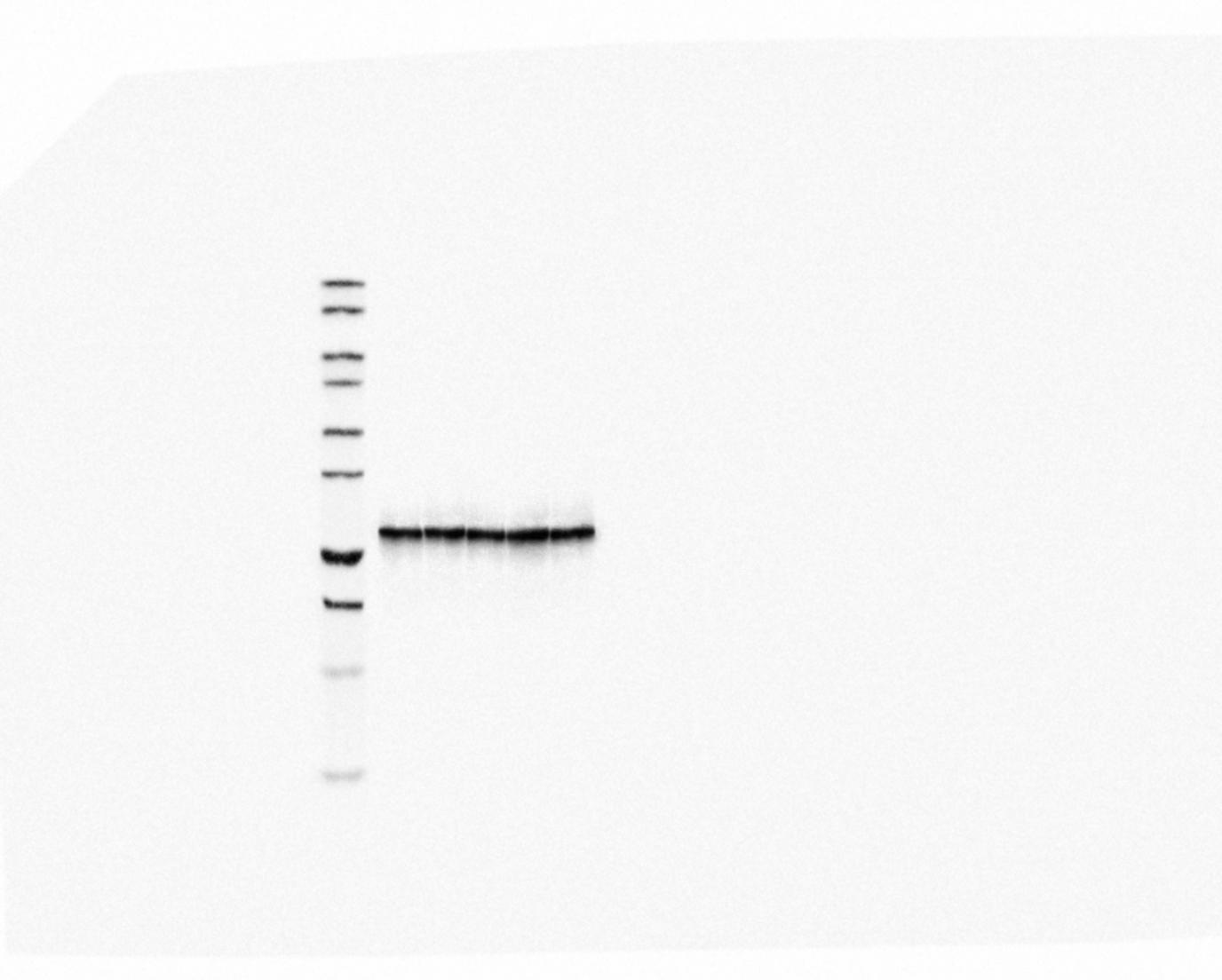

Supplement: Supplementary file 2 [file Data_Sheet_2.zip › Original Western Blot Images for Manuscript EPI-S2024-0015.R2/β-actin-1.jpg]

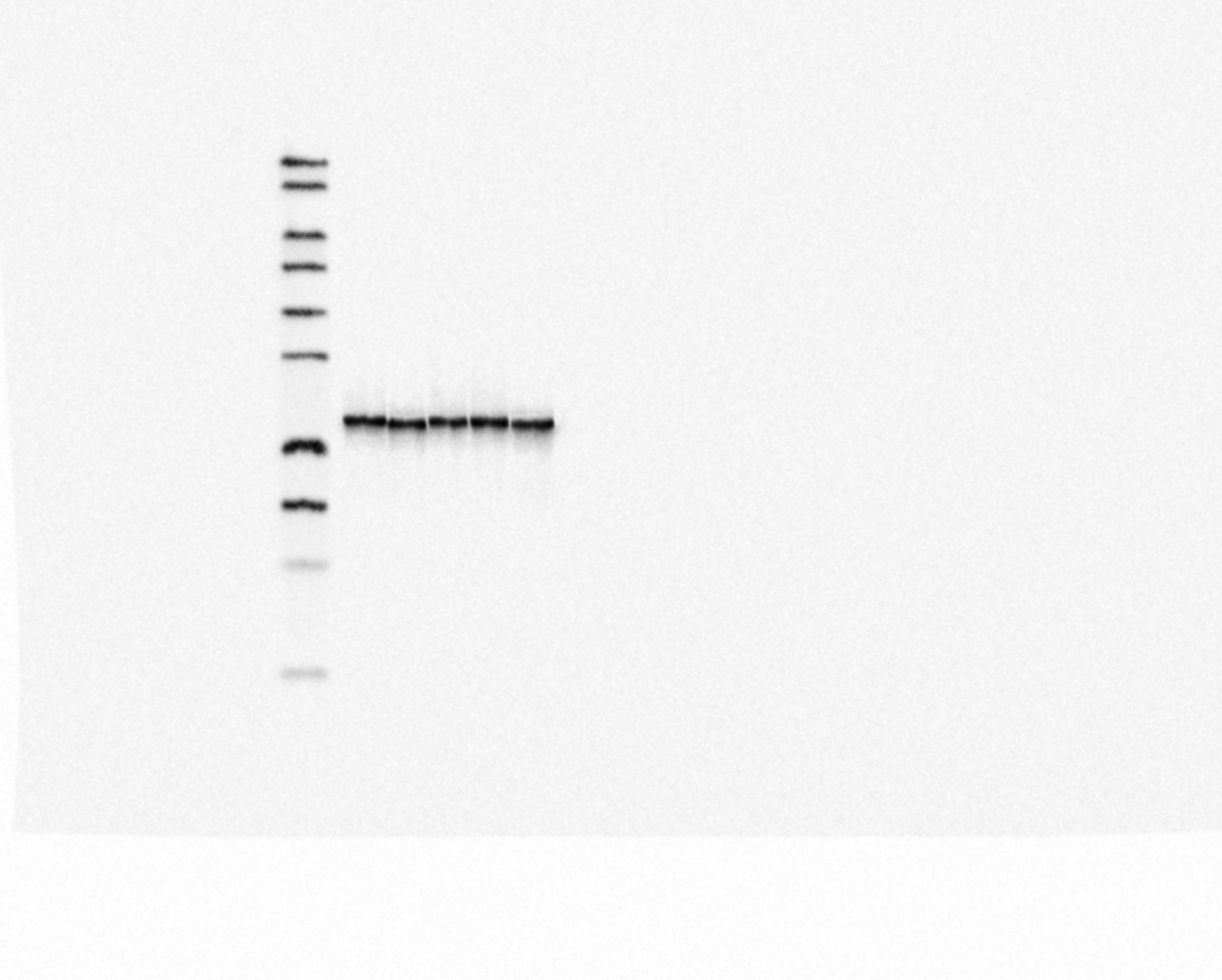

Supplement: Supplementary file 2 [file Data_Sheet_2.zip › Original Western Blot Images for Manuscript EPI-S2024-0015.R2/β-actin-2.jpg]

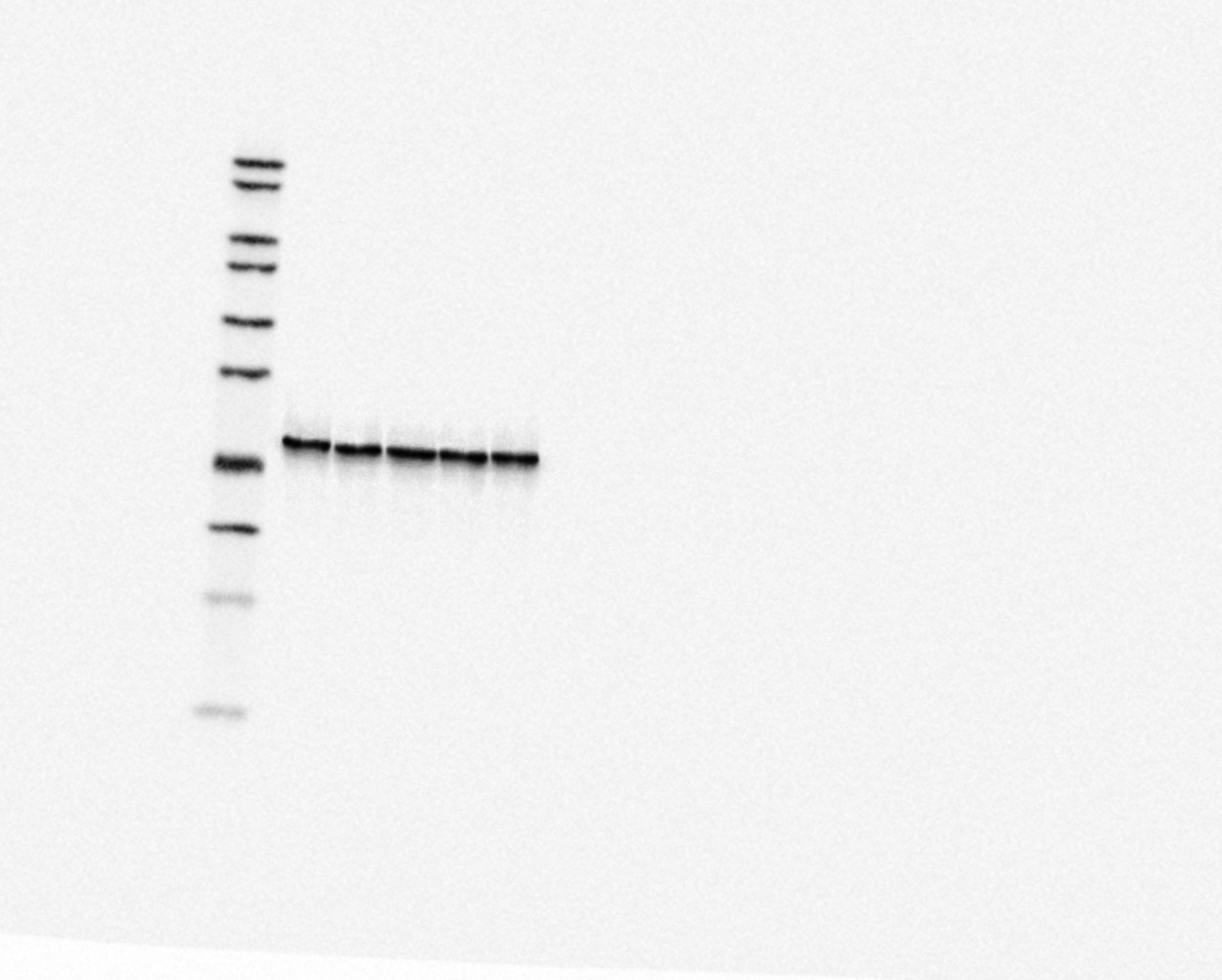

Supplement: Supplementary file 2 [file Data_Sheet_2.zip › Original Western Blot Images for Manuscript EPI-S2024-0015.R2/β-actin-3.jpg]
